# Synthesis of UDP-apiose in Bacteria: The marine phototroph *Geminicoccus roseus* and the plant pathogen *Xanthomonas pisi*

**DOI:** 10.1371/journal.pone.0184953

**Published:** 2017-09-20

**Authors:** James Amor Smith, Maor Bar-Peled

**Affiliations:** 1 Complex Carbohydrate Research Center (CCRC), University of Georgia, Athens, GA, United States of America; 2 Dept. of Biochemistry and Molecular Biology, University of Georgia, Athens, GA, United States of America; 3 Dept. of Plant Biology, University of Georgia, Athens, GA, United States of America; Dong-A University, REPUBLIC OF KOREA

## Abstract

The branched-chain sugar apiose was widely assumed to be synthesized only by plant species. In plants, apiose-containing polysaccharides are found in vascularized plant cell walls as the pectic polymers rhamnogalacturonan II and apiogalacturonan. Apiosylated secondary metabolites are also common in many plant species including ancestral avascular bryophytes and green algae. Apiosyl-residues have not been documented in bacteria. In a screen for new bacterial glycan structures, we detected small amounts of apiose in methanolic extracts of the aerobic phototroph *Geminicoccus roseus* and the pathogenic soil-dwelling bacteria *Xanthomonas pisi*. Apiose was also present in the cell pellet of *X*. *pisi*. Examination of these bacterial genomes uncovered genes with relatively low protein homology to plant UDP-apiose/UDP-xylose synthase (UAS). Phylogenetic analysis revealed that these bacterial UAS-like homologs belong in a clade distinct to UAS and separated from other nucleotide sugar biosynthetic enzymes. Recombinant expression of three bacterial UAS-like proteins demonstrates that they actively convert UDP-glucuronic acid to UDP-apiose and UDP-xylose. Both UDP-apiose and UDP-xylose were detectable in cell cultures of *G*. *roseus* and *X*. *pisi*. We could not, however, definitively identify the apiosides made by these bacteria, but the detection of apiosides coupled with the *in vivo* transcription of bUAS and production of UDP-apiose clearly demonstrate that these microbes have evolved the ability to incorporate apiose into glycans during their lifecycles. While this is the first report to describe enzymes for the formation of activated apiose in bacteria, the advantage of synthesizing apiose-containing glycans in bacteria remains unknown. The characteristics of bUAS and its products are discussed.

## Introduction

Apiose (3-C-[hydroxymethyl]-D-erythrofuranose, Api) is a common sugar residue of the plant pectic polymers rhamnogalacturonan-II (RG-II) and apiogalacturonan in vascular plant species [1, 2]. In addition, apiose-containing small secondary metabolites were detected in lichens [3] and in the fungus *Morchella conica* [4].

**Fig 1:**
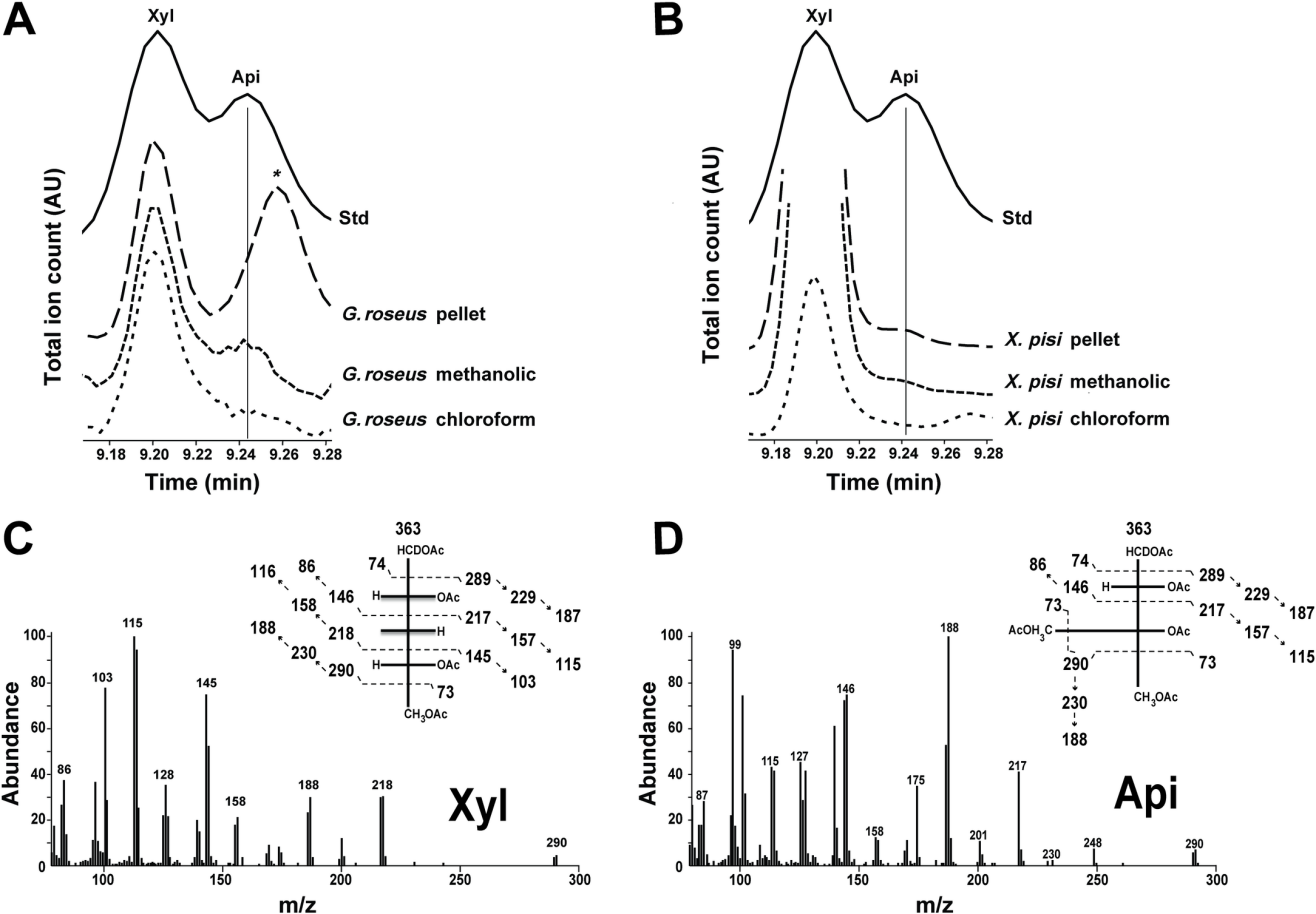
Detection of apiose in bacteria. GC-MS analysis of alditol-acetate derivatives from methanolic, chloroform and cell pellet fractions of *G*. *roseus* (A) and *X*. *pisi* (B). Standard (Std) contains authentic xylose and apiose. The region of the total ion count for xylose (Xyl) and apiose (Api) alditol-acetate derivatives is expanded. (B) and (C) are MS fragmentation patterns for standard Xyl and Api, respectively. * indicates unidentified residue.

In a screen for novel bacterial glycans, we unexpectedly found an apiosyl residue in methanolic extracts of two gram-negative proteobacteria: the alphaproteobacteria *Geminicoccus roseus* and the gammaproteobacteria *Xanthomonas pisi* (Fig 1). In addition to apiose we also detected xylose but the latter has been previously described in certain bacteria [5–11].

In plants the activated nucleotide sugar donor used to synthesize apiose-containing glycans is UDP-apiose (UDP-Api). A bifunctional enzyme (UAS) converts UDP-glucuronic acid (UDP-GlcA) predominantly to UDP-Api but UDP-xylose (UDP-Xyl) is also made in a ratio close to 2:1. The UAS enzyme was characterized in several plant species and was named UDP-apiose/UDP-xylose synthase [12–15]. UAS belongs to the short-chain dehydrogenase/reductase (SDR) superfamily, which also includes UDP-xylose synthase (UXS) [16]. UDP-xylose synthase (UXS) was identified in plant, human and bacteria, and this enzyme has a single activity converting UDP-GlcA to UDP-Xyl. Functionally characterized bacterial UXSs [10, 11] belong to nitrogen fixing rhizobium species that symbiotically reside with plants. Another bacterial species that harbors UXS is the common human gut microbial species belonging to *Bacteroides* [11]. Certain plant pathogenic bacteria like *Ralstonia solanacearum* also synthesize UDP-Xyl as a byproduct of the related enzyme Rsu4kpxs [9]. Lastly, the common gram-negative bacterial gene ArnA encodes a UDP-GlcA decarboxylase able to form an intermediate UDP-4-keto-L-arabinose [9, 17] on route to the formation of UDP-4-amino-4-deoxy-L-arabinose and UDP-4-deoxy-4-formamido-L-arabinose. Interestingly, ArnA can also produce UDP-Xyl as a byproduct [18]. None of the UXS enzymes were demonstrated to have dual activity like UAS.

In an effort to explain the existence of apiose in these bacteria (*G*. *roseus* and *X*. *pisi)*, we searched their genomes for potential genes encoding UAS activity. Using plant UAS as a probe, we identified UAS-like homologous genes from several bacterial species. We show in this report that they are capable of synthesizing UDP-Api from UDP-GlcA. The origin of UAS in these bacteria remains unclear.

## Results

### Detection of apiose in extracts of *G*. *roseus* and *X*. *pisi*

Bacteria grown in liquid and agar media were collected and subjected to methanolic extractions. Following chemical hydrolyses, conversion of the monosaccharides to their alditol-sugar derivatives and GC-MS analyses, the methanolic extract revealed a peak that migrated like apiose (Fig 1). The GC chromatogram and the electron impact MS fragmentation pattern of this peak structure was not reported in Bacteria but had all the chromatographic and mass spectral features of an apiose. For example, the peaks at *m/z* 290 (Fig 1A, insert) suggest a cleavage between C2-C3 of an apiose-derivative. The peaks at *m/z* 248 and 247 are likely secondary ion fragments due to loss of *m/z* 42 (ketene) from *m/z* 290 and 289, respectively. Peaks at *m/z* 229/230 and 187/188 are due to loss of *m/z* 60 (acetate) and loss of acetate and ketene (*m/z* 60 + 42) from *m/z* 289/290, respectively. The major peak at *m/z* 188 represents the deuterated form of the acetylated apiose-derivative. No apiose was detected in the organic solvent extracts. By contrast, a small amount of Api was apparent in the cell pellet of *X*. *pisi* but not *G*. *roseus* (Fig 1). In addition to Api, these extracts also consist of other sugar residues like glucose, galactose, fucose, rhamnose, arabinose, and xylose (Xyl). While both Xyl and Api have similar structures their MS fragmentation patterns differ significantly (Fig 1C).

Because mass spectra are not sufficient to discriminate apiose from its two potential epimers, we sought to further validate the nature of the apiose-like GC-MS peak by identifying genes involved in the formation of the activated sugar.

### Identification and phylogeny analysis of bacterial UDP-apiose/UDP-xylose synthase-like homologs

The BLink and BLAST programs [19] were used to identify bacterial proteins in the NCBI non-redundant database that share sequence similarity to functional bacterial UXSs as well as plant UAS. A search in Bacteria for homologs to the amino acid sequence of *Arabidopsis* AXS/UAS1 (AEC08054.1) identified several candidates in proteobacteria: *Candidatus entotheonella*, *Geminicoccus roseus*, *Xanthomonas pisi* and *Yangia pacifica* with 43, 46, 48 and 49% sequence identity to *Arabidopsis* AXS/UAS1, respectively. These bacterial UAS-like gene homologs are named herein bUAS.

**Fig 2:**
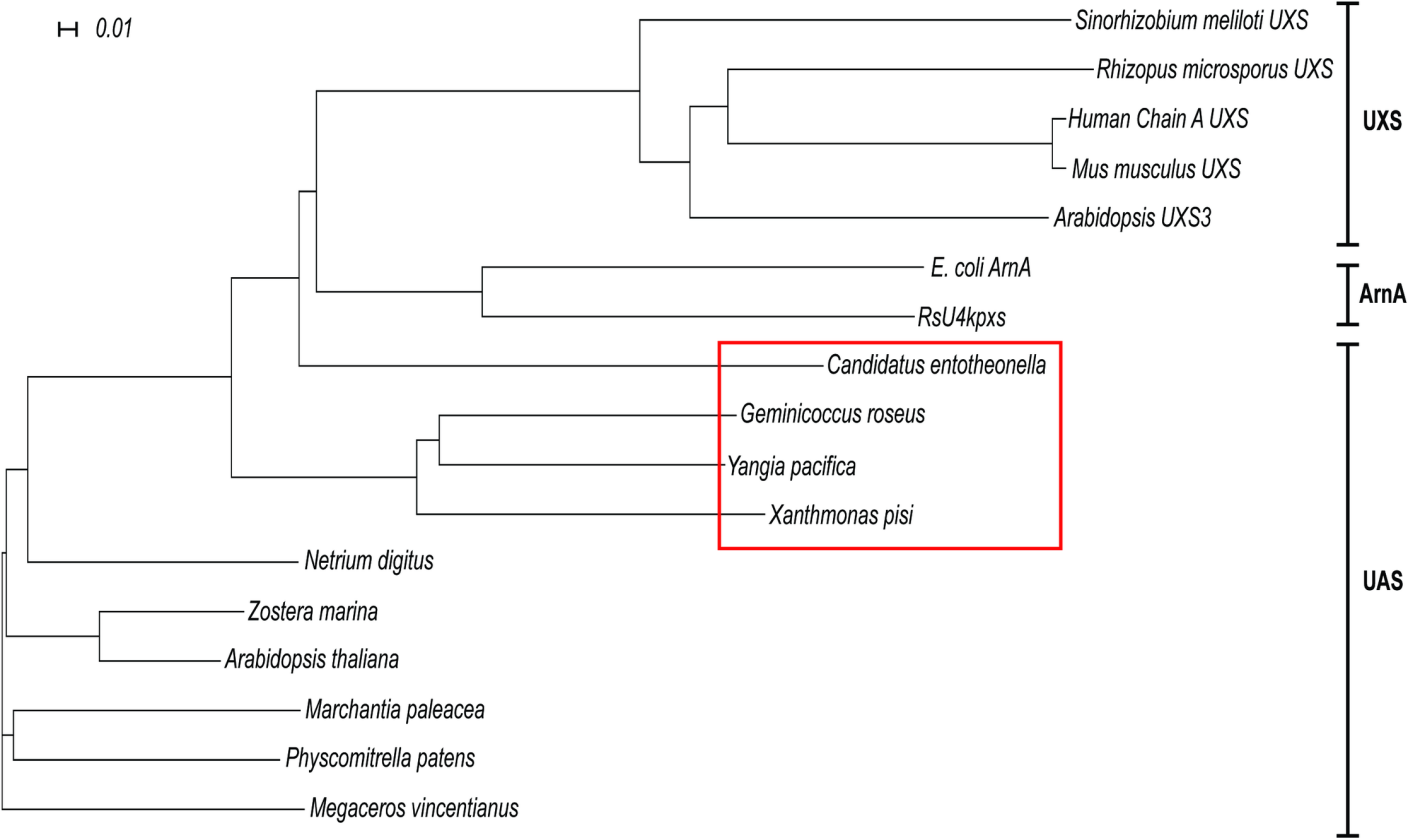
Phylogeny of bUASs. Phylogenetic analysis of proteins involved in the synthesis of UDP-apiose (UAS) and UDP-xylose (UXS). Amino acid sequences used are the C-terminal region of *Escherichia coli* ArnA (WP_032205568.1) that forms UDP-4-keto-arabinose, *Ralstonia solanacearum* UDP-4-keto-pentose/UDP-xylose synthase (RsU4kpxs, WP_011001268.1), UXSs from bacteria (*Sinorhizobium meliloti*, ACY30251.1) mammal (human & *Mus musculus*, NP_079352.2 & NP_080706.1), fungi (*Rhizopus microsporus*, CEI96046.1) and plant (*Arabidopsis* UXS3; NP_001078768.1). The bacterial UAS-like sequences used are from *Candidatus entotheonella*, *Geminicoccus roseus*, *Xanthomonas pisi* and *Yangia pacifica* (ETX00953.1, WP_084506503.1, WP_084725965.1 and WP_066111466.1). Other UASs used are from green algae (*Netrium digitus*, AOG75413.1), from hornwort (*Megaceros vincentianus*, AOG75412.1), from liverwort (*Marchantia paleacea*, AOG75410.1) from moss (*Physcomitrella patens*, AOG75414.1), and from angiosperms (*Arabidopsis thaliana & Zostera marina*, KMZ68719.1 & NP_180353.1). Bacterial UAS are outlined by a red box. Alignment was made using Clustal Omega [24–26] and the tree generated using Dendroscope [20].

An unrooted phylogenetic tree (Fig 2) was generated using Dendroscope [20]. The analysis compared an alignment of amino acid sequences of functional plant and microbial proteins belonging to the short-chain dehydrogenase/reductase (SDR) family with the amino acid sequences of the above bacterial UAS (bUAS) proteins. The PRALINE sequence alignment [21] (S1 Fig) included UDP-xylose synthases (UXSs) from plant, mammal and fungi, and two bacterial enzymes; a bifunctional UDP-4-keto-pentose/UDP-xylose synthase (RsU4kpxs) from the plant pathogen *Ralstonia solanacearum* and the C-terminal portion of ArnA that has a UDP-glucuronic acid 4-oxidase-6-decarboxylase activity [9, 17]. UAS, UXS, RsU4kpxs and ArnA are all decarboxylases that contain domains common to all SDRs: a conserved N-terminal Gly-*X-X*-Gly-*X-X*-Gly motif (S1 Fig; X = any amino acid) that is proposed to be involved in NAD^+^ binding and Tyr-*X-X-X*-Lys motif with an upstream Ser that forms the catalytic site of the SDR family [16, 22, 23]. The bUASs cluster into a group distinct from the clades for bacterial ArnA and the UXSs (Fig 2).

BLAST [19] analyses show *X*. *pisi* UAS (XpUAS) protein has 65% sequence identity with the homolog from *G*. *roseus* (GrUAS), 64% with that from *Y*. *pacifica* (YpUAS) and 44% with that from *C*. *entotheonella* (CeUAS). The closest plant homolog to XpUAS belongs to *Ornithogalum longibracteatum* (sea onion; AMM04380.1) and shares 51% sequence identity to the XpUAS. Similarly, the GrUAS is 47% identical to UAS from the sea grass *Zostera marina*, and YpUAS is 50% identical to UAS from *Vitis vinifera* (XP_002270884.1). Because no sea sponge genomes or transcriptomes are available, we cannot infer any relation of CeUAS to that of its host *Theonella swinhoei*, but its closest plant UAS homolog belongs to *Amborella trichopoda* (XP_006851788.1) with 44% sequence identity. Examination of the genes flanking bUASs in each of these bacteria revealed that each is flanked by different genes, with no apparent conserved operon.

Employing the phylogeny to assess origins of the bUASs was inconclusive. While the bUASs share a branch in the phylogeny (Fig 2), they have varying sequence identities and no common theme. Additionally, the bUAS sequences are not very identical to plant UAS sequences. It is possible that bUASs originated from a bacterial ancestral gene source, however we cannot exclude the possibility that bUASs came from plant UAS through a gene transfer event. The limited number of bUAS examples prohibits specifying their origin or the evolutionary advantage they confer. Future deposition of additional bacterial sequences will provide a better understanding of the relationship among UASs.

To date there are no reports that bacteria produce apiose [1]. Because we could not determine at the GC-MS level if the pentose was truly apiose or an apiose epimer (for example two possible epimer forms of apiose at C-2 and C-3), and because the metabolic pathway leading to formation of these apiose-like residues was unknown, we decided to clone the genes and determine if the UAS-like homologs are capable of converting UDP-GlcA to UDP-Api, or perhaps utilizing other UDP-sugar uronates, for example UDP-*N*-acetyl-glucosaminuronic acid (UDP-GlcNAcA) or UDP-galacuronic acid (UDP-GalA). To this end, XpUAS, GrUAS and CeUAS were cloned, expressed in *E*.*coli* and then functionally characterized.

### Cloning of bUAS and in microbe formation of UDP-apiose

**Fig 3:**
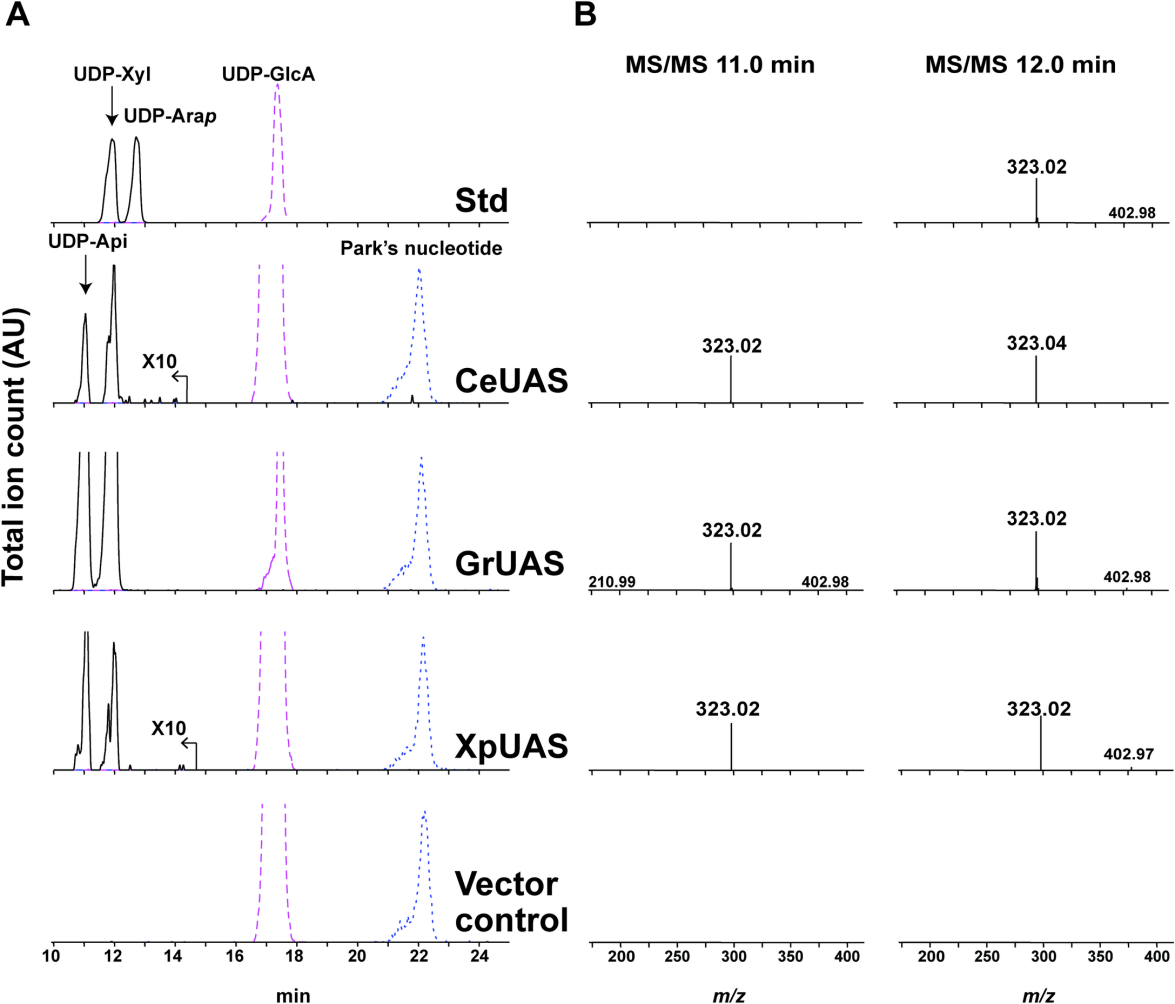
Activity of recombinant bacterial UDP-apiose synthase (bUAS) by in microbe assay. Analysis of in microbe nucleotide sugars by HILIC-LC-ESI-MS/MS. (A) top panel elution of standard (Std): UDP-GlcA, UDP-Xyl and UDP-arabinopyranose (UDP-Ara*p*); Nucleotide sugars were extracted from *E*. *coli* cells induced to express genes encoding CeUAS, GrUAS, XpUAS or empty vector as the control (bottom panel). [M-H]^-^ ions diagnostic for UDP-pentose (*m/z* 535.0, solid line), UDP-hexuronic acid (*m/z* 579.0, dashed line) and Park’s nucleotide (*m/z* 595.6, dotted line) are displayed. Park’s nucleotide is a UDP-MurNAc-pentapeptide that is used as an internal standard for nucleotide-sugar detection as it is abundantly made in *E*. *coli*. The *m/z* signal for CeUAS and XpUAS is amplified by a factor of 10. (B) Second stage MS fragmentation data for the peaks at the indicated retention times; Left column 11.0 min and right column 12.0 min. MS/MS ions at *m/z* 323.0, 211.0, 403.0 are consistent with predicted fragmentation of a UDP-sugar into [UMP-H]^-^, [Ura-2H]^-^, and [UDP-H_2_O-H]^-^, respectively.

The coding sequences of the selected UAS homologs were cloned into a modified pET28b *E*. *coli* expression vector [27]. The bUAS-containing plasmids or empty plasmid (negative control) were then individually transformed into *E*. *coli* together with a pCDF plasmid containing the UDP-glucose dehydrogenase [28] that provides the potential UDP-GlcA substrate for bUAS *in vivo*. Nucleotide sugar-containing extracts from the isopropyl β-D-thiogalactoside (IPTG)-induced *E*. *coli* cells were chromatographed by hydrophilic interaction liquid chromatography (HILIC) and analyzed by electrospray ionization tandem mass spectrometry (ESI-MS/MS) in the negative mode. Two peaks eluting at 11.0 and 12.0 min (Fig 3A) were observed in strains harboring the bUAS but not in *E*. *coli* cells harboring plasmid control. The mass spectra (Fig 3B) of both peaks showed an [M-H]^-^ ion at *m/z* 535.0, that gave MS/MS ion fragments at *m/z* 403.0, 323.0 and 211.0 which are consistent with [UDP-H_2_O-H]^-^, [UMP-H]^-^ and [Ura-2H]^-^, respectively. The *m/z* 535.0 was not found in control *E*. *coli* expressing empty plasmid. Proton NMR (^1^H NMR) analyses confirmed that the UDP-pentose eluting at 11.0 min was UDP-Api, and not UDP-apiose-epimer. These data suggest that the bUAS enzymes do synthesize UDP-Api.

### Characterization of purified recombinant bUAS

**Fig 4:**
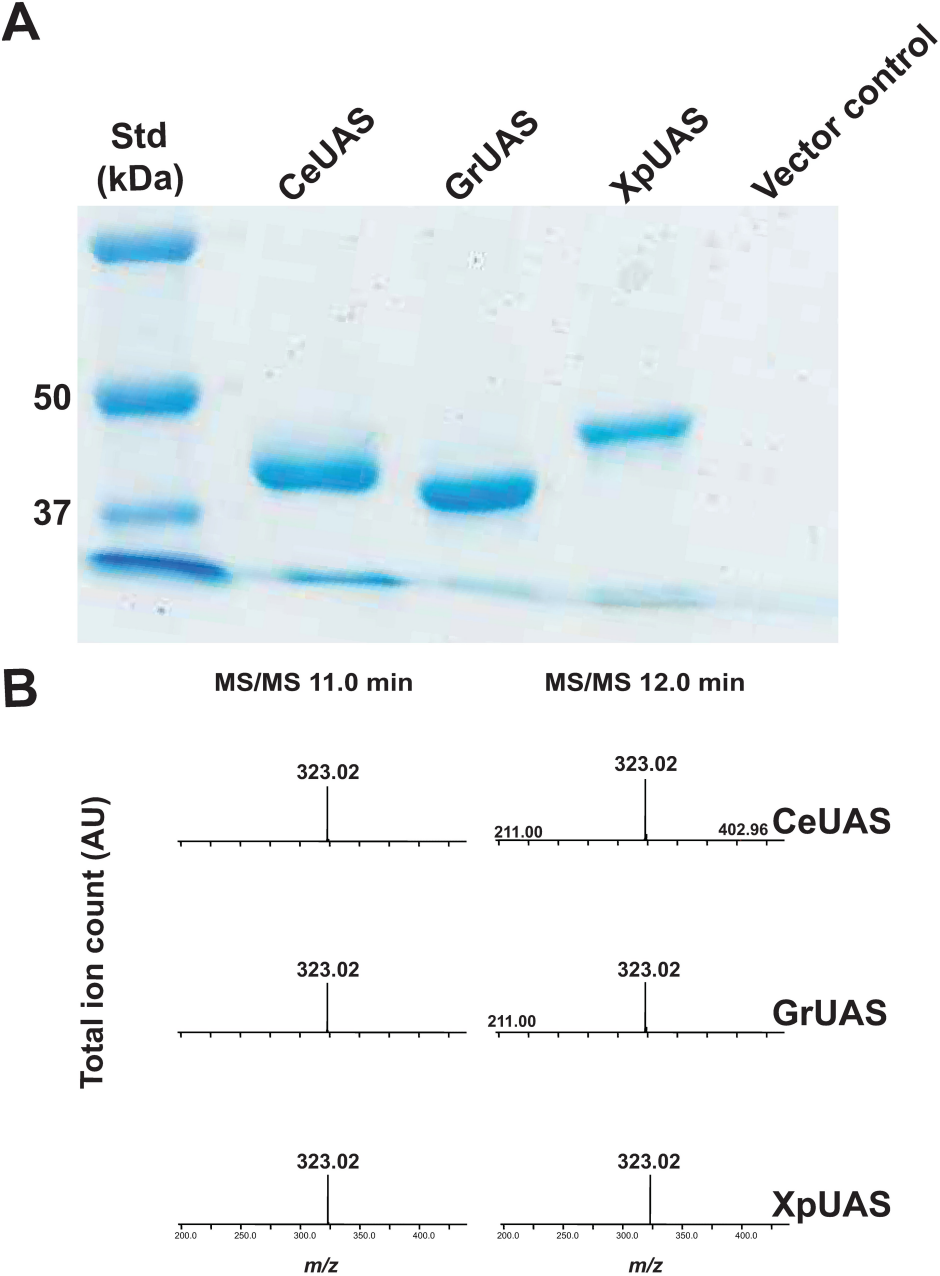
Activity of purified recombinant bUAS proteins. (A) Nickel-purified proteins from *E*. *coli* cells induced to express CeUAS, GrUAS, XpUAS and empty vector control with expected sizes of CeUAS, GrUAS and XpUAS: 44.2, 42.9 and 45.1 kDa, respectively. (B) MS/MS for the *m/z* 535.0 peaks of UDP-Api (left column, elution time 11.0 min) and UDP-Xyl (right column, 12.0 min). MS/MS ions at *m/z* 323.0, 211.0, 403.0 are consistent with predicted fragmentation of a UDP-sugar into [UMP-H]^-^, [Ura-2H]^-^, and [UDP-H_2_O-H]^-^, respectively.

**Fig 5:**
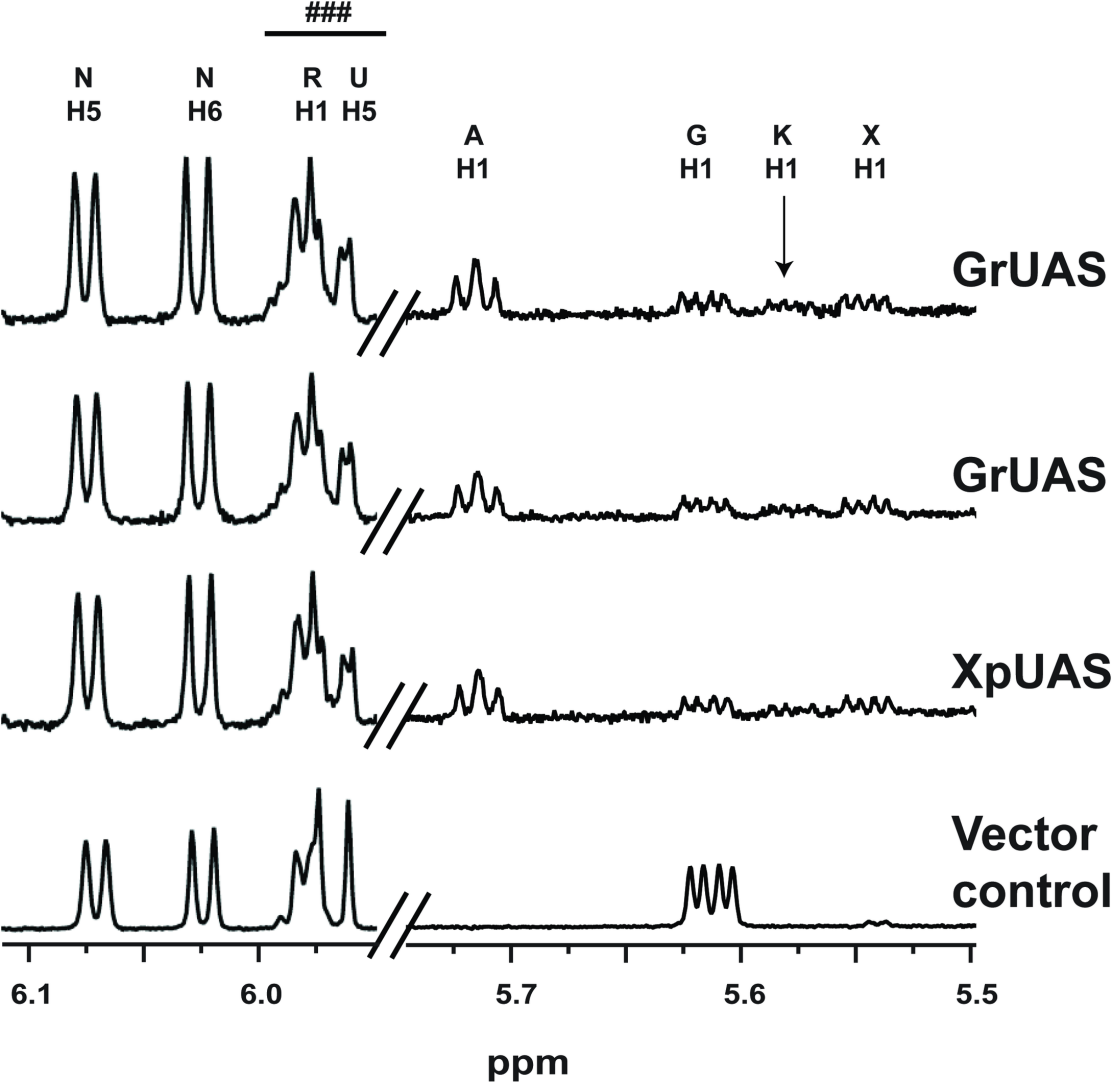
^1^H NMR spectra of purified recombinant bUAS reactions. Selected regions of ^1^H NMR spectra diagnostic for the products and intermediates generated by incubation of UDP-GlcA and NAD^+^ with the purified recombinant UASs from bacteria. Anomeric region between 5.50 and 5.75 ppm for the H1 protons of UDP-GlcA (G), UDP-Api (A) and UDP-Xyl (X) products and UDP-4-keto-Xyl (K) intermediate are shown. NMR region (5.95 and 6.15 ppm) diagnostic for UDP and NAD^+^ cofactor is included. NMR spectral traces from top to bottom show UAS activity of CeUAS, GrUAS, XpUAS and empty vector control. Peaks labeled N correspond to H5 and H6 protons of NAD^+^. ### indicates a mixture of ribose (R) and uracil (U) proton peaks of UDP from substrate and products. For additional chemical shift assignments see S1 Table.

To obtain additional evidence for the nucleotide sugar metabolism and the specific UDP-sugar uronate that the bUASs are utilizing to form UDP-Api, the recombinant His_6_-tagged proteins were solubilized from *E*. *coli* cells and purified using nickel-affinity column. The recombinant bUASs migrated on SDS-PAGE with a predicted mass of between 43 and 45 kDa (Fig 4A). Each purified UAS was shown by HILIC-ESI-MS/MS to convert UDP-GlcA to two UDP-pentose products in the presence of NAD^+^. MS/MS analysis (Fig 4B) of these product peaks (11.0 and 12.0 min) also gave fragment ions at *m/z* 323.0 that is consistent with [UMP-H]^-^. Anomeric (H-1) peaks consistent with the presence of UDP-Api and UDP-Xyl were detected in all the ^1^H NMR spectra when the recombinant enzyme assays were performed in deuterated buffer (Fig 5).

**Fig 6:**
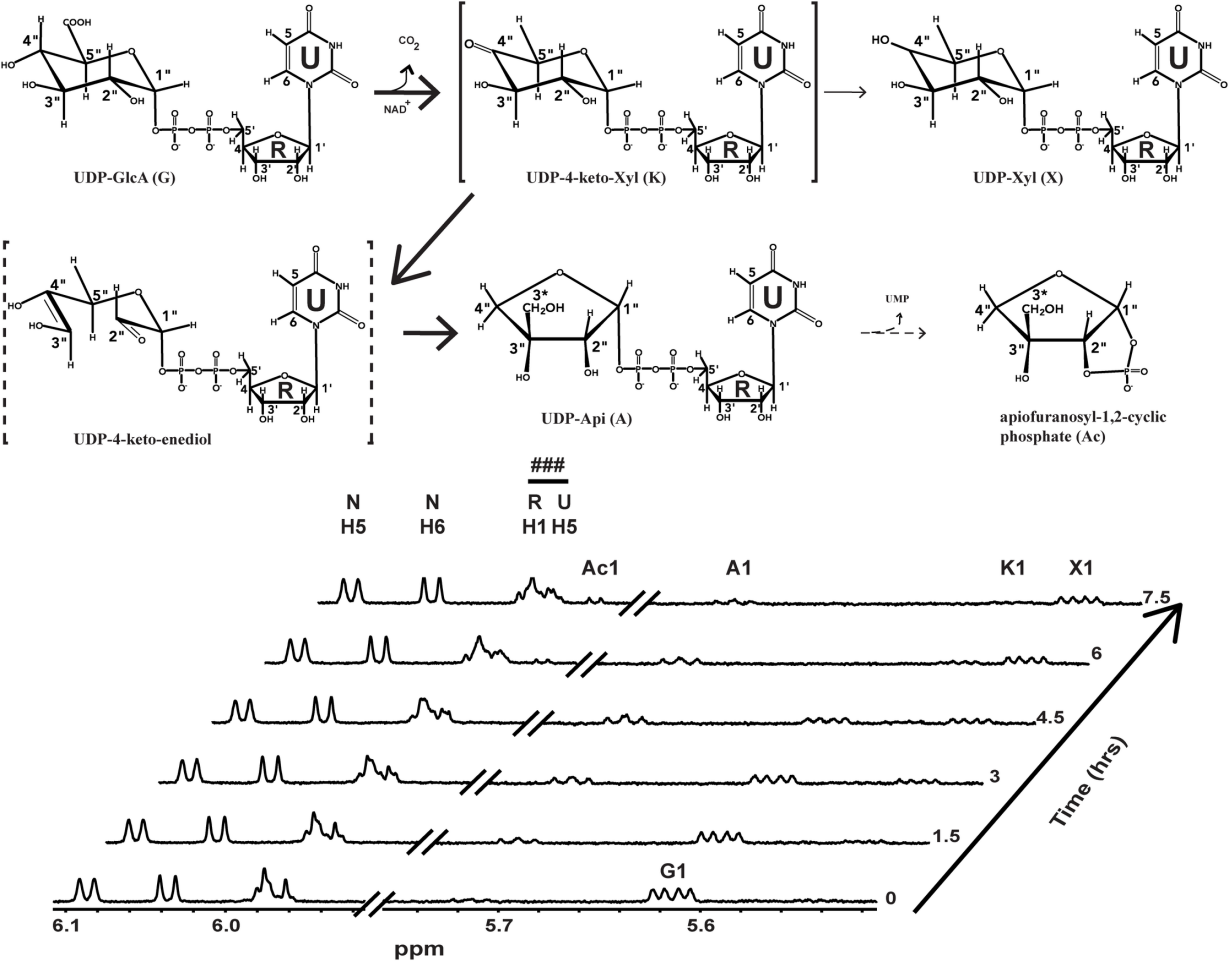
UAS reaction mechanism based on real time NMR analysis of recombinant GrUAS activity. NMR spectra of the UDP-apiose synthase activity at 37 ^o^C showing conversion of the substrate (UDP-GlcA), to intermediate (UDP-4-keto-Xyl), products (UDP-Api and UDP-Xyl ~ 2:1 ratio) and degradation product (apiofuranosyl-1,2-cylcic phosphate, Ac). The proton NMR spectrum of the sugar anomeric regions (H-1”s, between 5.5 and 6.1 ppm) of substrate, intermediate and products is shown. Only select time-resolved spectra are displayed to prevent overcrowding of peaks. ### indicates a mixture of ribose (R) and uracil (U) proton peaks of UDP from substrate and products. For additional chemical shift assignments see S1 Table.

GrUAS was the most highly expressed protein and was thus selected for further characterization. Real time ^1^H NMR spectroscopic analysis of the products formed when GrUAS reacts with UDP-GlcA (Fig 6 and S1 Table) confirmed that UDP-Api is the first product formed. GrUAS produces UDP-Api and UDP-Xyl in a ratio of ~1.7: 1.0, which is similar to characterized plant UASs [29–31]. The NMR study with GrUAS also confirmed that some of the UDP-Api is degraded and converted to the apiofuranosyl-1,2-cyclic phosphate during the *in vitro* reaction (Fig 6); this instability of UDP-apiose is a known phenomenon [29, 31, 32]. No degradation of UDP-Xyl is discernible over the course of the reaction.

**Fig 7:**
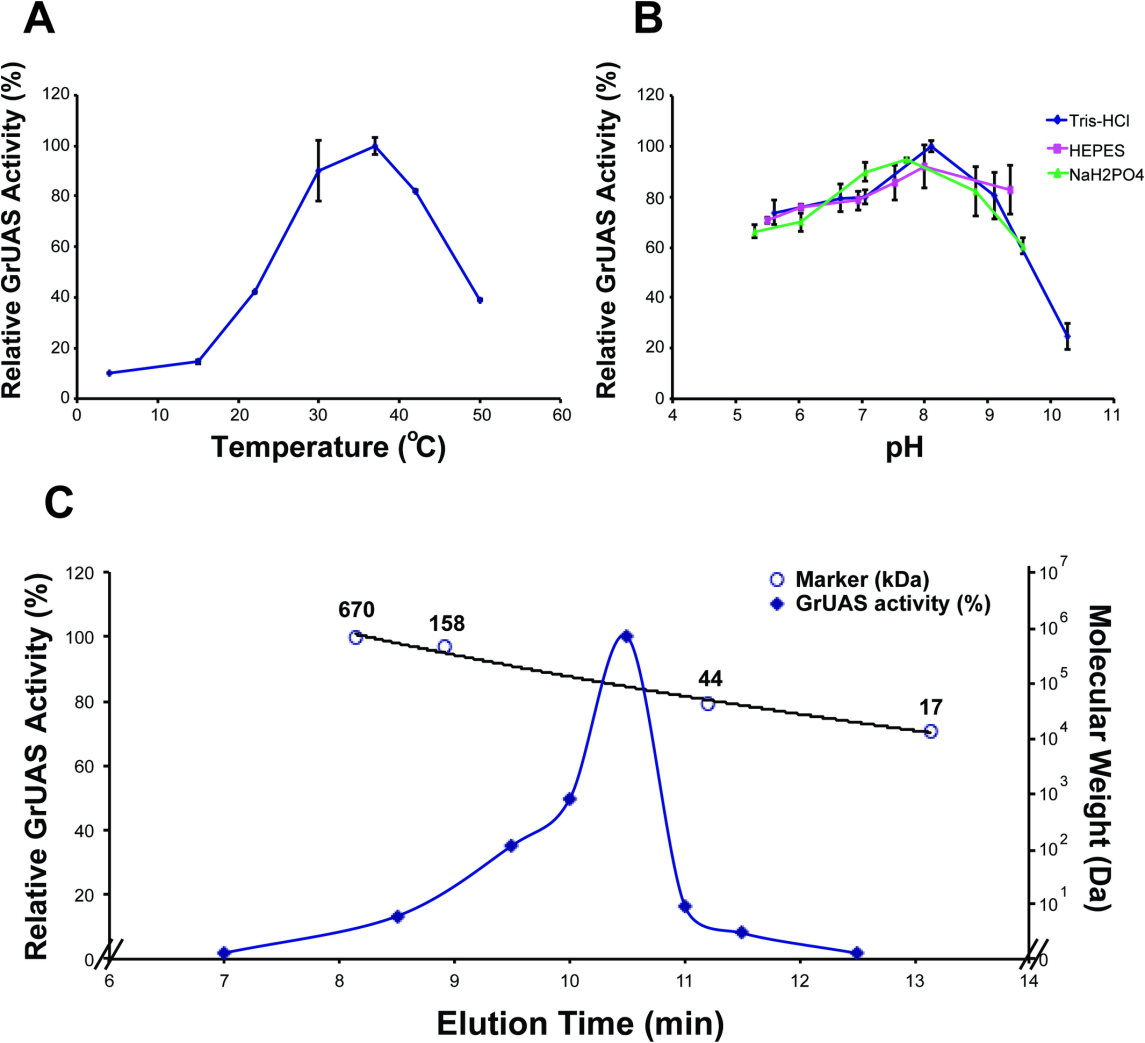
Characterization of GrUAS. The effects of temperature and pH on the activity of recombinant GrUAS. (A) Maximum activity of GrUAS is at 37°C. (B) Maximum activity of GrUAS is in Tris-HCl at a pH of 8.1. (C) Size-exclusion chromatography suggests recombinant GrUAS is active as dimer. The relative activity (indicated by closed diamonds) was determined by HPLC. The molecular weight of the enzyme in solution is based on the relative elution times of standard protein markers (indicated by open circles).

The recombinant GrUAS is most active in 50 mM Tris-HCl, pH 8.0–8.5, at 37°C (Fig 7A and 7B) and exists in solution as a dimer with a predicted size of 84 kDa (Fig 7C). Table 1 shows that GrUAS has a *Km* of 251 μM similar to that for *Spirodela* UAS [31], and a Kcat/*Km* of 60.2 nM s^-1^, while recombinant *Arabidopsis* AXS/UAS1 has a reported *Km* of 7 μM and Kcat/*Km* of 43 nM s^-1^ [30]. Previous studies have shown that UAS is inhibited by certain nucleotides and nucleotide sugars, especially UDP-Xyl and UDP-GalA [30, 31]. Table 2 demonstrates that under our assay conditions UDP-Xyl and UDP-GalA inhibited GrUAS activity by 9% and 77%, respectively.

**Table 1:** Enzymatic properties of recombinant GrUAS.

	GrUAS
Optimal pH^a^	7.7–8.1
Optimal Temperature (°C)^a^	37–42
*K*_m_ (μM)^b^	251 ± 44.6
*V*_max_ (nM-s^-1^)	70.4 ± 4.53
*k*_cat_ (s^-1^)	15.1 ± 0.97
*k*_cat_ /*K*_m_ (nM-s^-1^)	60.2 ± 7.05
Mass of Active Protein/Dimer (kDa)^c^	(84.3)

^a^Optimal pH was determined using different buffers (Tris-HCl, sodium phosphate, & HEPES; pH range 5.3–10.3). Temperature assays were conducted in Tris-HCl buffer pH 7.9.

^b^The data presented are calculated from the average of three experiments.

^c^The mass of active GrUAS eluted from Superdex75 gel filtration column (10.53 min) was estimated based on extrapolation of standard protein marker.

**Table 2:** Effect of nucleotide sugars and nucleotides on recombinant GrUAS activity.

Additive	Relative activity (%)^a^
Water (Control)	100 ± 4.8
UDP-glucose	89.1 ± 5.0
UDP-galactose	98.5 ± 4.2
UDP-arabinose	88.5 ± 5.6
UDP-xylose	91.4 ± 5.6
UDP-galacturonic acid	33.4 ± 9.5
UDP	72.6 ± 5.5
UMP	92.6 ± 5.0
GDP	85.2 ± 7.9
GMP	94.3 ± 3.7
CDP	85.3 ± 4.6
CMP	93.8 ± 4.5
TDP	85.0 ± 4.8
TMP	87.9 ± 4.9
NADP	79.6 ± 3.3
NADPH	82.6 ± 0.9
NADH	105.3 ± 1.9

^a^Amounts of unreacted UDP-GlcA were determined by HPLC. The activity is calculated as the average relative amount of UDP-GlcA consumed compared to the control from three experiments.

### Transcript analysis of bUAS and detection of UDP-apiose in *X*. *pisi* and *G*. *roseus* cultures

**Fig 8:**
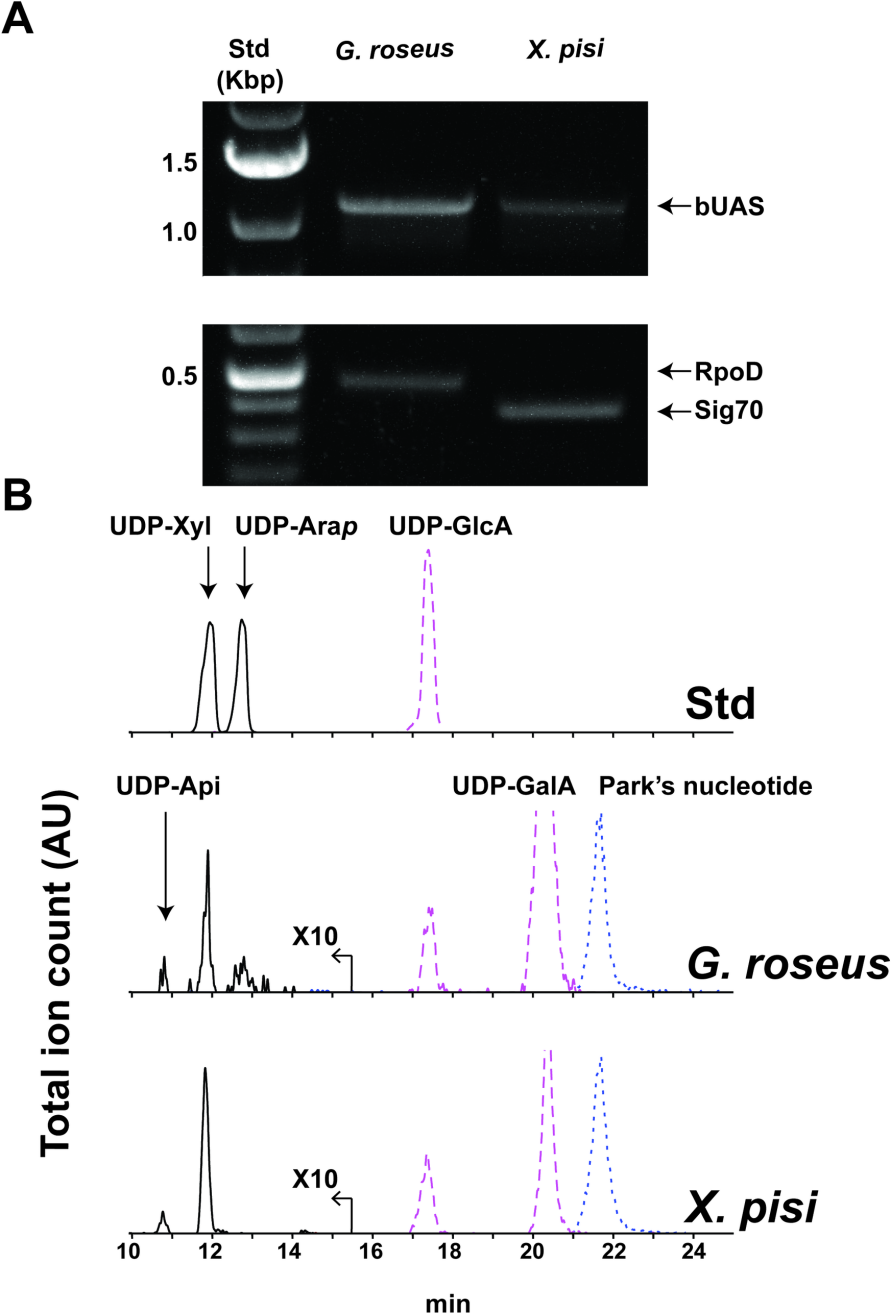
Detection of bUAS transcripts and UDP-apiose. *In vivo* indication for the functional activity of bUAS genes and enzymes. (A) RT-PCR analyses showing bUAS transcripts of *G*. *roseus* and *X*. *pisi*. (B) HILIC-LC-ESI-MS/MS analysis of aq-methanolic (MeOH:chloroform:H_2_O; 40:40:20, v/v/v) extracts. Negative mode [M-H]^-^ ions diagnostic for UDP-pentose (*m/z* 535.0, solid line; amplified by a factor of 10), UDP-hexuronic acid (*m/z* 579.0, dashed line) and Park’s nucleotide (*m/z* 595.6, dotted line) are displayed.

To investigate if the bUASs are transcribed in these bacteria and if they produce UDP-Api *in vivo*, cultures of *X*. *pisi* and *G*. *roseus* were grown in liquid and agar media. Analysis of RNA for bUAS transcript was positive for both species (Fig 8A) albeit at lower amounts when compared with the control transcripts; *G*. *roseus* sigma factor RpoD (WP_027134046.1) and *X*. *pisi* Sig70 (EGD11293.1). Furthermore, at culture steady state, small amounts of UDP-Api and UDP-Xyl were detected by HILIC-LC-ESI-MS/MS in aqueous extracts of living culture (Fig 8). Together, these data demonstrate that genes encoding bacterial UAS are expressed and the UAS enzymes are functionally active *in vivo* leading to production of UDP-Api in the specific bacteria tested.

## Materials and methods

### Bacterial strains

*Geminicoccus roseus* ATCC BAA-1445 (*G*. *roseus)* and *Xanthomonas pisi* ATCC 35936 (*X*. *pisi*) were obtained from the American Type Culture Collection in Manassas, VA, USA. *G*. *roseus* strain was isolated from a marine aquaculture system in Germany [33], and *X*. *pisi* strain was isolated from *Pisum sativum* in Japan [34]. Unless otherwise stated, *G*. *roseus* cultures were grown on marine agar (BD Difco, Franklin Lakes, NJ, USA) at 30 ^o^C, and *X*. *pisi* cultures grown on nutrient agar (BD Difco) at 25 ^o^C. Liquid cultures were maintained in 125 mL of either marine broth or nutrient broth, shaking at 250 rpm.

### Glycosyl residue composition analysis

5 to 7-day-old cultures (30 ml) were centrifuged (10,000 g, 5 min, 4°C) and cell pellets suspended in 10 volumes of cold MeOH:chloroform:H_2_O (40:40:20, v/v/v). The suspensions were transferred to 15 ml falcon tubes and vortexed for 10 min (30 sec every 2 min, 4°C). The suspensions were centrifuged (10,000*g*, 5 min, 4°C) and separated into a top methanolic-water phase (termed methanolic), medial interphase (termed pellet) and bottom organic chloroform phase. A portion (20 μl) of the top methanolic fraction was analyzed on HILIC-LC-ESI-MS/MS (see below) and the remainder transferred to a 13 mm borosilicate tube. The bottom chloroform fraction was transferred to a separate tube. The remaining interphase was re-suspended in 2 ml distilled deionized water (DDW) and samples centrifuged (10,000 g 5 min, 4°C). Supernatant was vacuum aspirated, the pellet was again re-suspended in 1 ml DDW and transferred to a new 13 mm borosilicate tube.

The methanolic and organic solvent extracts or cell pellets (~1 mg) were supplemented with myo-Inositol (10 μl of 5 mM solution) as an internal standard, evaporated to dryness at room temperature using a stream of air (REACTIVAP III, Thermo Fisher, Waltham, MA, USA) and then hydrolyzed for 1 h at 120°C with 1 ml of 1 M TFA. TFA was removed by evaporation under a stream of air (40°C) and the residue washed with isopropanol (3 x 500 μl). The released monosaccharides were then converted into their corresponding alditol-acetate derivatives according to York *et al* [35], and the final residue dissolved in acetone (100 μl).

A fraction (1 μl) of each of the alditol-acetate derivative samples was analyzed by gas-liquid chromatography (GLC; 7890A, Agilent, Santa Clara, CA, USA) equipped with a mass selective detector (EI-MS, Agilent 5975C) and separated over a RTx-2330 fused silica column (Restek, Bellefonte, PA, USA) as previously described [31]. Alditol-acetate derivatives of standard apiose, rhamnose, fucose, ribose, arabinose, xylose, mannose, glucose, and galactose (50 μg each) were prepared under the same conditions as samples. Monosaccharides were identified based on their retention times and their electron impact (EI) mass spectra.

### Identification and cloning of CeUAS, GrUAS and XpUAS

The BLASTP program [19] and BLAST Link (BLink) tool were probed to identify bacterial proteins (taxid: 2) in the NCBI non-redundant database that share amino acid sequence homology to the *Arabidopsis* AXS/UAS1 (NP_180353.1). Analyses of hypothetical proteins belonging to the proteobacteria *Candidatus entotheonella*, *Geminicoccus roseus* and *Xanthomonas pisi* revealed that they share homology to UXS. The predicted protein for XpUAS lacked 38 amino acids at the N-terminal region based on sequence alignment with other UASs. To obtain the entire XpUAS ORF, the *X*. *pisi* whole genome shotgun sequence (NZ_JPLE01000032) was used to extend the nucleotide sequence to include 114 nucleotides upstream of the predicted transcript. The nucleotide sequences corresponding to the *C*. *entotheonella* and *G*. *roseus* proteins and the extended full-length nucleotide sequence for predicted XpUAS were used for primer design and cloning.

Genomic DNA (gDNA) was isolated from 5-day-old liquid cultures (3 ml) of *G*. *roseus* and *X*. *pisi*. Cells were pelleted (14,000 rpm, 1 min) and 200 μl extraction buffer [0.2 M Tris pH 8, 0.25 M NaCl, 25 mM ethylenediaminetetraacetic acid (EDTA), 1% SDS] added, and the samples were vortexed for 2 min. Samples were spun down (12,000 rpm, 5 min), and 150 μl of supernatant was transferred to a new tube. An equal volume of isopropanol was added, and precipitated gDNA was pelleted (12,000 rpm, 5 min). Supernatant was vacuum aspirated and samples were allowed to dry under laminar flow hood for 20 min. gDNA was re-suspended in 100 μl TE (10 mM Tris pH 8, 1 mM EDTA) and stored at 4°C.

A portion of gDNA (2 μl), dNTP’s (0.2 mM) and 1 unit of Phusion® high-fidelity DNA polymerase (New England Biolabs, Ipswich, MA, USA) were mixed with 0.2 μM of each forward and reverse primers (IDT, Coralville, IA, USA; S2 Table) and used to amplify GrUAS and XpUAS under the following thermal cycler conditions: one 98°C denaturation cycle for 30 s followed by 25 cycles (each of 8 s denaturation at 98°C; 25 s annealing at 60°C; 30 s elongation at 72°C), and finally termination at 4°C. The PCR product was directly cloned into the *E*. *coli* expression vector pET28b modified to contain an N-terminal His_6_ tag followed by a TEV cleavage site [27].

Because no axenic monoculture of *C*. *entotheonella* was available, a synthetic ORF gene corresponding to the nucleotide sequence of CeUAS was obtained (GenScript, Piscataway, NJ, USA). The ORF was cloned into the modified pET28b expression vector [27], using forward and reverse primers (see S2 Table).

Following cloning of the individual bUAS genes, the plasmids were sequence verified (Georgia Genomics Facility, Athens, GA, USA) and termed, pET28b-TEV-CeUAS.1, pET28b-TEV-GrUAS.1 and pET28b-TEV-XpUAS.1. Their amino acid sequences were deposited in GenBank™ (accession numbers MF191704, MF191705 and MF191706, respectively).

### Analysis of nucleotide sugars produced in microbe

Nucleotide sugars from *E*. *coli* harboring the expression plasmids were harvested as described [31, 36]. BL21-derived *E*. *coli* cells (3 or 60 ml) co-transformed with pCDFDuet-BtbDH and either pET28b-TEV-CeUAS.1, pET28b-TEV-GrUAS.1, pET28b-TEV-XpUAS.1 or empty pET28b vector control were grown in LB medium [1.0% (w/v) Bacto tryptone, 0.5% (w/v) Bacto yeast extract, and 1.0% (w/v) NaCl] supplemented with 35 μg/ml chloramphenicol, 50 μg/ml kanamycin, and 25 μg/ml spectinomycin at 37°C and 250 rpm, induced with Isopropyl β-D-thiogalactoside (IPTG, 0.5 mM) at an OD_600_ of 0.6 and grown at 30°C for 4 h. In microbe nucleotide sugars were extracted and analyzed by hydrophilic interaction liquid chromatography electrospray ionization tandem mass spectrometry (HILIC-LC-ESI-MS/MS) as described [31].

### His_6_-tagged protein expression and purification

BL21-derived *E*. *coli* cells were transformed with pET28b-TEV-CeUAS.1, pET28b-TEV-GrUAS.1, pET28b-TEV-XpUAS.1, or the empty vector control. The cells were grown at 37°C and 250 rpm for 16 h in 20 ml LB media containing kanamycin (50 μg/ml) and chloramphenicol (35 μg/ml). A portion (5 ml) of the culture was transferred to fresh 245 ml LB supplemented with antibiotics and grown under the same conditions until its OD_600nm_ was 0.8. IPTG (0.5 mM) was then added to induce expression of the gene, and the culture then grown for an additional 4 h at 30°C and 250 rpm. The induced cultures were cooled on ice and centrifuged (6,000 g, 10 min, 4°C). The cell pellet was suspended in 10 ml lysis buffer [50 mM Tris-HCl pH 7.6, 10% (v/v) glycerol, 1 mM EDTA, 5 mM dithiothreitol 0.5 mM phenylmethylsulfonyl fluoride]. The cells were ruptured by sonication and proteins were then isolated after centrifugation as described [27]. The final soluble protein fraction (Fraction S20) was collected and kept on ice prior to immediate purification.

The different His_6_-tagged proteins, including control empty plasmid, were each purified using fast-flow Ni-Sepharose (2 ml resin packed in a 15 x 1 cm polypropylene column; GE Healthcare, Chicago, IL, USA) as previously described [31], where the purified His_6_-bUAS-protein eluted in fraction E7 and had activity. The active enzymes were dialyzed (6,000–8,000 molecular weight cut-off; Spectrum Laboratories Inc, Rancho Dominguez, CA, USA) at 4°C three times for a total of 2 h against 50 mM Tris-HCl pH 7.6, containing 0.15 M NaCl, 10% (v/v) glycerol, 1 mM DTT, and 10 μM NAD^+^. The dialysates were divided into 150 μμl aliquots, flash-frozen in liquid nitrogen and stored at -80°C. Aliquots of purified protein were assayed for activity and analyzed on SDS-PAGE.

SDS-PAGE was performed with 12% (w/w) polyacrylamide gels. Proteins were stained with 0.1% (w/v) Coomassie Brilliant Blue R-250 in aqueous (aq.) 20% methanol (MeOH) containing 7% (v/v) acetic acid and de-stained with aq. 20% methanol containing 7% (v/v) acetic acid. Protein concentrations were determined with the Bradford reagent [37] using bovine serum albumin (BSA) as standard, and the molecular mass of active recombinant GrUAS was estimated by size-exclusion chromatography as previously described [31].

### Recombinant bUAS enzyme assays

Unless otherwise indicated, the 50 μl reactions were performed in 50 mM Tris-HCl pH 7.9, containing 1 mM NAD^+^, 1 mM UDP-GlcA, and up to10 μg of purified protein. The assay mixtures were incubated at 37°C for up to 45 min and the reactions terminated by placing the tubes in boiling water for 2 min followed by the addition of an equal volume of chloroform. The suspensions were vortexed and centrifuged (12,000 g, 5 min, 22°C), and the aqueous phase analyzed for nucleotide sugars. ^1^H NMR assays were performed in deuterium oxide (D_2_O) using 30 μg purified protein in final volume of 180 μl.

### Characterization of recombinant GrUAS

GrUAS activity was assayed in different buffers, at different temperatures and with various additives and nucleotide sugars. For pH studies, reactions in total volume of 50 μl consisted of purified recombinant GrUAS (10 μg), 1 mM NAD^+^, 1 mM UDP-GlcA and various pH buffers (100 mM) and kept at 37°C for 30 min. Inhibition assays were performed by first supplementing the standard reaction mixtures with various nucleotides and nucleotide sugars, addition of purified protein and incubation. The amounts of reactants and products were determined by UV spectroscopy and used to calculate enzyme activity as follows. The products from each recombinant enzyme assay were chromatographed over a column (200 x 1 mm) packed with 15 μm Source 15Q anion exchange resin (GE Healthcare) by elution with a linear gradient (5 mM to 0.6 M) of ammonium formate over 25 min at a flow rate of 0.25 ml/min using an Agilent 1100 Series HPLC equipped with an G1313A auto-sampler, a G1315B diode array detector, and ChemStation software. Nucleotides and nucleotide sugars were detected by their A_261nm_ (for UDP-sugars) and A_259nm_ (for NAD^+^). The concentrations of reactants and products were determined by comparison of their peak areas to a calibration curve of standard UDP-GlcA [9].

Selected kinetic parameters of recombinant GrUAS (10 μg) were determined by varying the concentrations of UDP-GlcA in 50 μl reactions consisting of 1 mM NAD^+^ in 50 mM Tris-HCl pH 7.9. Reactions were kept for 7 min at 37°C quenched with an equal volume of chloroform and then vortex mixed. The reaction products in the aqueous phase were separated using a Q-15 anion exchange column as described above and reaction rates calculated from the depletion of the UDP-GlcA signal integral normalized to the NAD^+^ signal integral. Values from three independent replicates were used to generate a non-linear regression plot and resultant data using GraphPad Prism Version 7.

### HILIC-LC-ESI-MS/MS

ESI-MS/MS analysis was performed on a Shimadzu (Kyoto, Japan) LC-ESI-MS-IT-TOF operating in the negative ion mode. Methanolic extracts, in microbe and recombinant enzyme assay products were mixed with 2/3 volume aq. 95% acetonitrile (ACN) containing 25 mM ammonium acetate and an aliquot (10–20 μl) chromatographed over an Accucore amide-HILIC column (150 x 4.6 mm; Thermo), eluted at 0.4 ml min^-1^ with a linear gradient of aq. 75% (v/v) acetonitrile containing 40 mM ammonium acetate, pH 4.4, to 50% (v/v) acetonitrile containing 40 mM ammonium acetate, pH 4.4, over 35 min using a Shimadzu LC-30AD HPLC. Mass spectra (mass range 100–2,000 *m/z*) were collected every 1.3 sec for 30 minutes. Second stage MS/MS data was collected by collision-induced dissociation (CID) with a collision energy of 35% and a nebulizing nitrogen gas flow of 1.5 ml min^-1^ (59).

### Real-time ^1^H and NMR enzyme assays

All spectra were obtained using a Varian Inova 600 MHz spectrometer equipped with a 3 mm cryogenic probe. Continuous ^1^H and 2-D HSQC NMR spectroscopic monitoring of reactions (180 μl volume) were carried out at 37°C in a mixture of D_2_O/H_2_O (9:1, v/v) containing 0.83 mM 2,2-dimethyl-2-silapentane-5-sulfonate (DSS, internal reference), 50 mM Tris-HCl, pH 7.9, 1 mM UDP-GlcA or ^13^C_6_-labeled UDP-GlcA, 1 mM NAD^+^ and purified recombinant enzyme. One-dimensional ^1^H NMR spectra with the water resonance signal pre-saturated were collected 5 minutes post addition of enzyme in order to optimize spectrometer settings, and then spectra were continuously averaged every 2.5 min for up to 8 h. All chemical shifts are referenced to DSS at 0.00 ppm [29].

### RNA isolation and RT-PCR

RNA was extracted [38] from a 5-day-old culture of *G*. *roseus* and a 2-day-old culture of *X*. *pisi* grown in liquid media. A 3 ml portion of liquid culture was centrifuged (14,000 rpm, 1 min, 22°C); supernatant discarded; and the cells were flash frozen in liquid nitrogen and stored at -80°C until extraction. Cell pellets were re-suspended in 400 μl TE-lysozyme (20 mM Tris-HCl, pH 8, 1 mM EDTA, 1 mg/ml lysozyme; Sigma L6876), vortexed at room temperature for 10 min; and extraction was carried out after addition of 40 μl of fresh 10X EB (0.3 M NaOAc, pH 5.2, 5% sarkosyl, w/v, 50 mM EDTA, 10% β-mercaptoethanol, v/v) and incubation at 65°C and mixing for 3 min. Subsequently, 440 μl of preheated acidic phenol was added, and samples incubated at 65°C while mixed by vortex for 7 min. Sample was then placed on ice for 3 min and centrifuged (10,000 g 5 min, 4°C). The top 350 μl of the aqueous phase was transferred to a new tube and an equal volume of chloroform added. Sample was vortexed for 1 min, incubated at room temperature for 7 min and then centrifuged (14,000 rpm, 5 min, 22°C). The top 200 μl of the aqueous phase was transferred to a new tube mixed with 200 μl of cold isopropanol and placed at -20°C overnight. Sample was then centrifuged (10,000 g, 10 min, 4°C) and the pellet was re-suspended in 75% ethanol, again centrifuged (10,000 g, 10 min, 4°C) and supernatant aspirated. Tubes were left open under laminar flow hood for 15 min to dry. Resulting nucleic acids were re-suspended in 40 μl sterile, deionized distilled water (DDW) and RNA concentration measured with a nanodrop (Thermo). To digest remnant genomic DNA, 2 μg of RNA was DNAse treated according to manufacturer guidelines (Thermo). Following DNA digest (37°C for 30 min) an equal volume of chloroform was added and mixed. Sample was centrifuged (14,000 rpm, 5 min, 22°C) and top aqueous phase transferred to a new tube. RNA (0.5 μg) was then reverse transcribed with a random hexamer primer (Thermo) using SuperScript III reverse transcriptase (Life Technologies; Carlsbad, CA, USA). A portion of the reverse transcriptase (RT) reaction (2 μl), dNTP’s (0.2 mM) and 1 unit of Phusion® high-fidelity DNA polymerase (New England Biolabs) were mixed with 0.2 μM of each forward and reverse primers (IDT; S2 Table) and used to amplify GrUAS, XpUAS, *G*. *roseus* sigma factor RpoD (GrRpoD) and *X*. *pisi* sigma factor 70 (XpSig70) with the following thermal cycler conditions: one 98°C denaturation cycle for 30 s followed by 25 cycles (each of 8 s denaturation at 98°C; 25 s annealing at 60°C; 30 s elongation at 72°C), and finally termination at 4°C.

## Discussion

This report is the first to describe the sugar apiose in Bacteria, and subsequently this study led us to identify functional genes responsible for synthesizing the activated donor UDP-apiose in prokaryotes. The activity of recombinant bacterial UDP-apiose synthase (bUAS) is specific and utilizes only UDP-glucuronic acid as a substrate.

The bacterial species appearing to contain a UAS were isolated from various sources, including soil and sea. The organization of the operons harboring bUAS in these bacteria is not conserved. The genes flanking bUAS are also not conserved. In contrast, the organizations of bacterial operons that carry out synthesis of other sugar nucleotides are conserved, for example dTDP-rhamnose [39–41]. Thus the ancestral origin of the bUAS remains somewhat elusive. Based on the current genomic database, to date, only 8 bUASs exist, but this number will likely increase as more marine bacteria are sequenced. Since the UAS belonging to the obligate endosymbiotic *C*. *entotheonella* shares less than 60% amino acid sequence identity with the other bUASs in this study, it is possible that there was no one single ancestral gene that gave rise to all of the bUASs. One possibility based on the limited sequences is that a duplication and sequence alteration of bacterial UXS or ArnA gave rise to these bUASs. However, one cannot rule out the possibility that early plant-derived UAS is the gene source for bUAS. Regardless of how or where they evolved, bUASs have the same catalytic domains and utilize the same chemistry as plant UASs (S1 Fig).

The bUASs are phylogenetically distinct from other short-chain decarboxylase/reductases (SDRs) and share a branch with functional plant UASs (Fig 2) All domains of life possess enzymes (UXSs) that convert UDP-GlcA to UDP-Xyl [10, 16, 42–44], while UAS appears to be limited to plants and now Bacteria. Only a select few prokaryotic species appear to contain apiose based on the presence of bUAS, suggesting it is advantageous for these organisms’ survival in their specific niche environments. Further support for this hypothesis is evidenced by the presence of UXS in the genomes of *G*. *roseus*, *C*. *entotheonella* and *Y*. *pacifica*, suggesting that these microbes require bUAS specifically for UDP-Api (not UDP-Xyl) synthesis.

The apiose residue observed in the methanolic extracts of *G*. *roseus* and *X*. *pisi* as well as the cell pellet fraction of *X*. *pisi* is likely to be incorporated as a secondary metabolite and potentially a cell wall glycan in *X*. *pisi*. Because no Api was detected in the chloroform-extracted fractions of culture, it is unlikely that the final Api residue is part of a glycolipid. Our previous work had shown that in green algae and basal land plants, Api residues also associated with secondary metabolites [31]. In vascular plants Api is present in the cell wall rhamnogalacturonan-II and apiogalacturonan and in secondary metabolites [1, 45–47]. It is therefore possible that these organisms share a related family of apioside metabolites.

The identification and functional characterization of bUAS reveals a new metabolic pathway of UDP-GlcA metabolism by which select microbes have adapted to compete within their local environments. Our results provide important tools for the future study of apiose and apiosides in bacteria. Continued study of the pathways leading to apiosylated metabolites in bacteria will uncover new and interesting glycosyltransferase activities valuable to carbohydrate research.

## Supporting information

S1 Fig UXS, ArnA, and UAS multiple sequence alignment. Full amino acid sequence alignment of the UDP-GlcA decarboxylase domain of *E*. *coli* ArnA (WP_032205568.1), bifunctional UDP-4-keto-pentose/UDP-xylose synthase from *Ralstonia solanacearum* (RsU4kpxs, WP_011001268.1), mouse & human UXS1 (MmUXS1 & hUXS1, NP_080706.1 & NP_079352.2), bacterial UXS from *Sinorhizobium meliloti* (SmUXS1, ACY30251.1), fungal UXS from *Rhizopus microsporus* (RmUXS, CEI96046.1), *Arabidopsis* AXS/UAS1 & UXS3 (AtUAS1 & AtUXS3, NP_180353.1 & NP_001078768.1), and UASs from the algae *Netrium digitus* (NdUAS, AOG75413.1), moss *Physcomitrella patens* (PpUAS, AOG75414.1), seagrass *Zostera marina* (ZmUAS, KMZ68719.1), hornwort *Megaceros vincentianus* (MvUAS, AOG75412.1), liverwort *Marchantia paleacea* (MpUAS, AOG75410.1), and the bacterial UASs from *Candidatus entotheonella* (CeUAS), *Geminicoccus roseus* (GrUAS), *Xanthomonas pisi* (XpUAS), and *Yangia pacifica* (YpUAS). Sequences were aligned with PRALINE [21] using the BLOSUM62 scoring matrix. Proposed catalytic and sites are indicated by outline and above the alignment.
(PDF)

S1 Table Chemical shifts and coupling constants for the protons of UDP-Api, UDP-Xyl and UDP-4-keto-Xyl formed from UDP-GlcA by recombinant GrUAS, and the apiofuranosyl-1,2-cyclic phosphate that is formed by spontaneous degradation of UDP-Api. ^a^ Chemical shifts are in ppm relative to internal DSS signal set at 0.00 ppm. Proton-proton coupling constants in Hz are shown as well as the J_1”, P_ coupling values between phosphate and the H1” proton of UDP-Api (A), UDP-Xyl (X) and UDP-4-keto-Xyl (K). The chemical shift values for uracil (U) and ribose (R) protons are similar to the uracil and ribose protons of other UDP-sugars. Compounds correspond to structures in Fig 6. * refers to the proton of the branched carbon in UDP-Api and apiofuranosyl-1,2-cyclic phosphate (Ac). Peak assignments were made according to known values [10, 29].
(DOCX)

S2 Table Primers used in plasmid generation, genotyping and transcript analysis. Obtained from IDT.
(DOCX)

## Abbreviations

Api: apiose
Xyl: xylose
GlcA: glucuronic acid
UAS: UDP-apiose synthase
HILIC: hydrophilic interaction chromatography
ESI: electrospray ionization
MeOH: methanol
ACN: acetonitrile
UXS: UDP-xylose synthaseRsU4kpxs
UDP-4-keto-pentose: UDP-xylose synthase
Gr: G. roseus
Ce: C. entotheonella
Xp: X. pisi
BtbDH: UDP-Glc dehydrogenase coding sequence from B. thuringiensis
IPTG: isopropyl -D-thiogalactoside
GalA: galacturonic acid
TEV: tobacco etch virus
EI: electron ionization
XIC: extracted ion chromatogram.
