# Supplementary material for: Synthesis of UDP-apiose in Bacteria: The marine phototroph Geminicoccus roseus and the plant pathogen Xanthomonas pisi
Source: PLoS One. 2017 Sep 20;12(9):e0184953. doi: 10.1371/journal.pone.0184953 (PMC5607165; doi:10.1371/journal.pone.0184953)
Supplement: S1 Fig — Full amino acid sequence alignment of the UDP-GlcA decarboxylase domain of E. coli ArnA (WP_032205568.1), bifunctional UDP-4-keto-pentose/UDP-xylose synthase from Ralstonia solanacearum (RsU4kpxs, WP_011001268.1), mouse & human UXS1 (MmUXS1 & hUXS1, NP_080706.1 & NP_079352.2), bacterial UXS from Sinorhizobium meliloti (SmUXS1, ACY30251.1), fungal UXS from Rhizopus microsporus (RmUXS, CEI96046.1), Arabidopsis AXS/UAS1 & UXS3 (AtUAS1 & AtUXS3, NP_180353.1 & NP_001078768.1), and UASs from the algae Netrium digitus (NdUAS, AOG75413.1), moss Physcomitrella patens (PpUAS, AOG75414.1), seagrass Zostera marina (ZmUAS, KMZ68719.1), hornwort Megaceros vincentianus (MvUAS, AOG75412.1), liverwort Marchantia paleacea (MpUAS, AOG75410.1), and the bacterial UASs from Candidatus entotheonella (CeUAS), Geminicoccus roseus (GrUAS), Xanthomonas pisi (XpUAS), and Yangia pacifica (YpUAS). Sequences were aligned with PRALINE [21] using the BLOSUM62 scoring matrix. Proposed catalytic and sites are indicated by outline and above the alignment. (PDF) [file pone.0184953.s001.pdf]

Unconserved 0 1 2 3 4 5 6 7 8 9 10 Conserved

```
           ..........10 ..........20 ..........30 ..........40 ..........50
CeUAS      ---------- ---------- ---------- ---------- ----------
GrUAS      ---------- ---------- ---------- ---------- ----------
XpUAS      ---------- ---------- ---------- ---------- ----------
YpUAS      ---------- ---------- ---------- ---------- ----------
RsU4kpxs   ---------- ---------- ---------- ---------- ----------
ArnA       ---------- ---------- ---------- ---------- ----------
AtUAS1     ---------- ---------- ---------- ---------- ----------
ZmUAS1     ---------- ---------- ---------- ---------- ----------
PpUAS      ---------- ---------- ---------- ---------- ----------
NdUAS      ---------- ---------- ---------- ---------- ----------
MvUAS      ---------- ---------- ---------- ---------- ----------
MpUAS      ---------- ---------- ---------- ---------- ----------
hUXS       MVSKALLRLV SAVNRRRMKL LLGIALLAYV ASVWGNFVNM RSIQENGELK
MmUXS      MVSKGLLRLV SSVNRRRMKL LLGIALFAYA ASVWGNFVNM RSIQENGELK
SmUXS      ---------- ---------- ---------- ---------- ----------
RmUXS      --------MS TTVGHKHVGS DVELSYL--- ---------- ----------
AtUXS3     ---------- ---------- ---------- ---------- ----------
Consistency 0000000000 0000000000 0000000000 0000000000 0000000000

           ..........60 ..........70 ..........80 ..........90 ..........100
CeUAS      ---------- ---------- ---------- ---------- ----MKILLL
GrUAS      ---------- ---------- ---------- ---------- ------MVIL
XpUAS      ---------- -MQRNPISQE ----RES--- ---------- ---VLKLIIL
YpUAS      ---------- -MR---IGTE ----NP---- ---------- ----MNIVIL
RsU4kpxs   ---------- ---------- ---------- ---------- ---MKKVLIL
ArnA       ------QTLG LVQGSRLNSQ PACTARR--- ---------- ---RTRVLIL
AtUAS1     ---------- MANGANRVDL -DGKPIQ--- ---------- ---PLTICMI
ZmUAS1     ---------- M---ANRVDL -DGRPIK--- ---------- ---TMTICMI
PpUAS      ---------- M---TARLNL -DGQEIK--- ---------- ---PLRICMI
NdUAS      ---------- MA-GGIRLDL -DGRPVA--- ---------- ---PLKICMI
MvUAS      ---------- MA---PRLDL -QGREIK--- ---------- ---PLRLCII
MpUAS      ---------- M---APRLDL -EGRCIQ--- ---------- ---PLKICII
hUXS       IESKIEEMVE PLR-EKIRDL EKSFTQKYP- -PVKFLSEK- -D-RKRILIT
MmUXS      IESKIEEIVE PLR-EKIRDL EKSFTQKYP- -PVKFLSEK- -D-RKRILIT
SmUXS      ---------- -MN-YFRNDF RGTLFGTNE- -ATDSLRRR- -SLQKRILVT
RmUXS      RDLYLPKEKN YVE-HKDSTI IYRHISSFP- -PVKQLSPS- -E-RKRILVT
AtUXS3     ---------- -----MAATS EKQNTTKPPP SPSPLRNSKF CQPNMRILIS
Consistency 0000000000 1110113243 0221223000 0000000000 0002458586

           GxxGxxG
           ..........110.........120.........130.........140.........150
CeUAS      GGGGFIGCHI TQKLLQST-D HLVNCYD--- ------LFDA RLQDSLGHS-
GrUAS      GCGGFVGSHL LDNLLRDESY LIEGWDP--- -------EDR KIRQHLDNP-
XpUAS      GCGGFVGSHL LDRLLSRSDM EIEGWD---- PD------AS KITKHIDDP-
YpUAS      GCGGFIGSHL LDRLLSTTDH KIVGWD---- PE------SS KIRKHLENP-
RsU4kpxs   GVNGFIGHHL SKRILESTDP EISQWE---- VY-GMDMQTE RLGDLVNHP-
ArnA       GVNGFIGNHL TERLLREDHY EVYGLD---- IG-SD----- AISRFLNHP-
AtUAS1     GAGGFIGSHL CEKLLTETPH KVLALD---- VYNDKIKHLL EPDTVEWS--
ZmUAS1     GAGGFIGSHL CEKLMDETLH TVIAVDVYND KI-RHLLEPA ETEGCAWS--
PpUAS      GAGGFIGSHL CEKLMETTKH SVLAID---- VC-GVKIQHL LALGQPWS--
NdUAS      GAGGFIGSHL CEALMWETHH TVIAID---- ---VSGDKIE HLLKEGSPWS
MvUAS      GAGGFIGSHL CEKLMWETEH SVLAID---- ---MYGEKIQ HLLTPSQAWS
MpUAS      GAGGFIGSHL CEALLWNTEH SILAVDI--- ----FCDKIQ HLLSPGEEWS
hUXS       GGAGFVGSHL TDKLMMD-GH EVTVVDN--- ---FFTGRKR NVEHWIGHE-
MmUXS      GGAGFVGSHL TDKLMMD-GH EVTVVDN--- ---FFTGRKR NVEHWIGHE-
SmUXS      GGAGFLGSHL CELLLGA-GH EVICLDN--- ---FSTGLRR NIAPLKRFD-
RmUXS      GGAGFVGSHL VDRLMWM-GH EVVVLDN--- ---FFTGTKR NVQHWIGHP-
AtUXS3     GGAGFIGSHL VDKLMENEKN EVVVADN--- ---YFTGSKE NLKKWIGHP-
Consistency *46**9*7*9 4769834336 5954481000 0001211234 4633243420
```

```
            .........160.........170.........180.........190.........200
CeUAS       -RFNYIHGDI RHDHTRVEKL IHD---ADVV VDLIAYANPS LYVSIPLDVF
GrUAS       -RFTLHRSIA N-APEALVEV ERAIRECDVV VNLAAICNPA DYNTRPLSVI
XpUAS       -RFTFHQVPC I-DDVELSRL EERLAEADAL VNLAAICNPA QYNTQPLDVI
YpUAS       -NFTLHQRYV D-DPETQQDL REAVAEGDVV INLAAICNPS EYNTNPISVI
RsU4kpxs    -RMHFFEGDI T-INKEWVEY HVR--KCDVI LPLVAIATPS TYVKAPLRVF
ArnA        -HFHFVEGDI S-IHSEWIEY HVK--KCDVV LPLVAIATPI EYTRNPLRVF
AtUAS1      GRIQFHRINI K-HDSRLEGL VKM---ADLI INLAAICTPA DYNTRPLDTI
ZmUAS1      GRIQFHRLNI K-HDSRLEGL IKM---SDLT INLAAICTPA DYNTRPLDTI
PpUAS       DRIEFYKINI K-SDTRLEGL IKV---SDLV INLAAICTPA DYNTRPLDTI
NdUAS       DRISFHAFNI R-HDSRLETF IRV---ADLT INLAAICTPA DYNTRPLDTI
MvUAS       DRIEFYKINI K-HDPRLEGL VKL---ADVT INLAAICTPA DYNTRPLETI
MpUAS       NRIDFFMINI K-HDPQTENL IKIS---DVV INLAAICTPA DYNTRPLDTI
hUXS        -NFELINHDV ------VEPL YIEV---DQI YHLASPASPP NYMYNPIKTL
MmUXS       -NFELINHDV ------VEPL YIEV---DQI YHLASPASPP NYMYNPIKTL
SmUXS       -TFRVIAHDV ------VEPI DLEV---DEI YNLACPASPP HYQADPIQTT
RmUXS       -HFELVRHDV ------VDPF MVEV---SQI YHLACPASPP HYQYNPTKTV
AtUXS3      -RFELIRHDV ------TEPL LIEV---DRI YHLACPASPI FYKYNPVKTI
Consistency 0665644358 2012225637 3441002957 66*87566*5 4*555*8577

                                            S
            .........210.........220.........230....:....240.........250
CeUAS       NLNFTENLKI TEYCVTHQ-- --------KR LIQFSTCEVY GKTVASL---
GrUAS       RANLFEVYPI VELCAKYKRW LVSFSTSETY GRTIASYLPA GTYDDP----
XpUAS       RANLLDSYPI VEACARAG-- --------TW LVYFSTSEVY GRTLSSY---
YpUAS       KSNLFDVYPI VELCVEYK-- --------RW LISFSTSECY GRTLSSYV--
RsU4kpxs    ELDFEANLPI VRSAAKYG-- --------KH LVFPSTSEVY GMCG------
ArnA        ELDFEENLRI IRYCVKYR-- --------KR IIFPSTSEVY GMC-------
AtUAS1      YSNFIDALPV VKYCSENN-- --------KR LIHFSTCEVY GKTIGSFLPK
ZmUAS1      YSNFIDALPV VKYCSEYA-- --------KR VIHFSTCEVY GKTVGSFLPK
PpUAS       YSNFVDALPV VQQCRDNG-- --------KR LIHFSTCEIY GKTIGSFLPR
NdUAS       HSNFVDALPV VRMCADYN-- --------KR LIHFSTCEVY GKTLANFLPR
MvUAS       YSNFIDALPV VRVCADNK-- --------KR LIHFSTCEVY GKTVGSFLPE
MpUAS       YSNFIDALPV VRYCTDN--- -------NKR LIHFSTCEVY GKTIGCFLPN
hUXS        KTNTIGTLNM LGLAKRV--- -------GAR LLLASTSEVY GDPE------
MmUXS       KTNTIGTLNM LGLAKRV--- -------GAR LLLASTSEVY GDPE------
SmUXS       KTCVIGSLNL LDLAARR--- -------GAR IFQASTSEIY GDPH------
RmUXS       KTSVMGTINM LGLAKRT--- -------KAR FLLTSTSEVY GDPE------
AtUXS3      KTNVIGTLNM LGLAKRV--- -------GAR ILLTSTSEVY GDPL------
Consistency 4675555757 7457553100 0000000066 7744995889 *453122110

                                                         YxxxK
            .........260.........270.........280.........290.....300
CeUAS       ----LQNQLP DHDNPAHAVF QEDNTAF--- ----ILGPVN KHRWIYSCAK
GrUAS       --------EL YELREDETPL IMGPIRN--- ----QRWT-- -----YACAK
XpUAS       ------VKTG SDDPDLYELR EEQTPLI-MG PVSNQRWT-- -----YACAK
YpUAS       GDTTYENPDL YELKEDETPL IMGPIVN--- ----QRWT-- -----YACAK
RsU4kpxs    ---------- -----DDEFD PEASPLV-YG PINKPRWI-- -----YACSK
ArnA        ---------- -----SDKYF DEDHSNLIVG PVNKPRWI-- -----YSVSK
AtUAS1      DHPLRDDPAF YVLKEDISPC IFGSIEK--- ----QRWS-- -----YACAK
ZmUAS1      DHPLRLQPEF YVLNENETPC IFGSIEK--- ----QRWS-- -----YACAK
PpUAS       DHPLKADPAF SVLKEDETAC IYGSIHK--- ----QRWS-- -----YACAK
NdUAS       DSLARQDPRY FVLKEDQTPY IYGPVEL--- ----QRWS-- -----YACAK
MvUAS       GSSLRKDKKY YLLKEDASPC IFGPIEK--- ----QRWS-- -----YACAK
MpUAS       DSPLR-KDNF YVLKEDESPC IFGSIDK--- ----QRWS-- -----YGCAK
hUXS        ---------- -VHPQSEDYW GHVNPIG--- ----PRAC-- -----YDEGK
MmUXS       ---------- -VHPQSEDYW GHVNPIG--- ----PRAC-- -----YDEGK
SmUXS       ---------- -VHPQVESYW GNVNPFG--- ----PRSC-- -----YDEGK
RmUXS       ---------- -EHPQKETYW GHVNPIG--- ----PRAC-- -----YDEGK
AtUXS3      ---------- -IHPQPESYW GNVNPIG--- ----VRSC-- -----YDEGK
Consistency 1001101111 1434545543 3345433000 0000595400 00000*557*
```

```
           ..........310.........320.........330.........340.........350
CeUAS       QLLERIIHAY GLEDRLNYTI IRPFNF--IG PRIDFLP-SE Q--------EG
GrUAS       QMTERLIYAH HDEEGLPFTI IRPLNF--FG PRMDYIPTRD G--------DG
XpUAS       QMMERIVYAH HSTSGMPFTI IRPLNF--FG ----------P RM------DYI
YpUAS       QMVERLVYAH HKEDGLPFTV VRPLNF--FG ----------P RM------DYI
RsU4kpxs    QLMDRVIWGY -GMEGLNFTL FRPFNW--IG PGLDSI-HTP K--------EG
ArnA        QLLDRVIWAY GEKEGLQFTL FRPFNW--MG PRLDNL-NAA R--------IG
AtUAS1      QLIERLVYAE GAENGLEFTI VRPFNW--IG ----------P RMDFIPGIDG
ZmUAS1      QLIERLIYAE GAENGLEFTI VRPFNW--IG ----------P RMDFIPGIDG
PpUAS       QLIERLIFGE GAENGMKFTI VRPFNW--IG ----------P RMDFIPGIDG
NdUAS       QLIERVIYGE SMENGLQFTI VRPFNW--IG ----------P RMDFIPGIDG
MvUAS       QLIERVIYAE GMENDLAFTI VRPFNWXLYP ----------W C--------GW
MpUAS       QLLERLVYAE GAENDLKFTI VRPFNW--IG PRMD------F IPGVDGPSHA
hUXS        RVAETMCYAY MKQEGVEVRV ARIFNT--FG ----------P R--------MH
MmUXS       RVAETMCYAY MKQEGVEVRV ARIFNT--FG ----------P R--------MH
SmUXS       RCAETLFFDF HKSHGVEIKI VRIFNT--YG ----------P R--------MR
RmUXS       RIAETLTYSY MRQEGVDVRV ARIFNT--FG ----------P R--------MS
AtUXS3      RVAETLMFDY HRQHGIEIRI ARIFNT--YG ----------P R--------MN
Consistency 8759776765 3465775668 6*68*50069 0000000005 6100000033

           ..........360.........370.........380.........390.........400
CeUAS       NP--------R VFS----HFL D----ALKTG EPMKLINGGH QRRTYTYIDD
GrUAS       VP--------R VLA----CFM A----ALLSG EPMQLVDGGQ ARRTIVSIYE
XpUAS       PRSDGEGIPR VLA----CFM A----ALLRG DPMKLVDGGQ ARRTIVAIEE
YpUAS       PQRDGDGVPR VLA----CFM G----ALLNG DPMKLVDGGK ARRTIVSIYE
RsU4kpxs    SS--------R VVT----QFL G----HIVRG ENIQLVDGGQ QKRAFTYVDD
ArnA        SS--------R AIT----QLI L----NLVEG SPIKLIDGGK QKRCFTDIRD
AtUAS1      PS--------E GVP----RVL ACFSNNLLRR EPLKLVDGGE SQRTFVYIND
ZmUAS1      PS--------E GVP----RVL ACFSNNLLRG EPLKLVDGGE SQRTFIYIKD
PpUAS       PS--------D SIP----RVL ACFSNSLMKG EPLKLVDGGK SQRTFIYIKD
NdUAS       PS--------D GVP----RVL ACFSTNLLRG EPLKLVDGGR SQRTFVYIKD
MvUAS       PQ--------R RCTKSPSMLQ ----HYLMRG EPLKLVDGGK SQRTFVYIKD
MpUAS       VP--------R VLA----CFS M----ALMKE EPLKLVDGGK AQRTFCYIKD
hUXS        MND------GR VVS----NFI L----QALQG EPLTVYGSGS QTRAFQYVSD
MmUXS       MND------GR VVS----NFI L----QALQG EPLTVYGSGS QTRAFQYVSD
SmUXS       PDD------GR VVS----NFI V----QALKG EDITIYGDGS QTRSFCFVED
RmUXS       PAD------GR VVS----NFI M----QAIRG ESLTLYGDGE QTRSFQYIHD
AtUXS3      IDD------GR VVS----NFI A----QALRG EALTVQKPGT QTRSFCYVSD
Consistency 4410000007 5750000366 4000047768 8686866*5 65*6746948

           ..........410.........420.........430.........440.........450
CeUAS       AVDCIVRIVE NPNQVCDKEI FNIGSPD-N- EISIRDLAFK MRCIYKRRWW
GrUAS       AVDAIRRVLE RP-ETSQNQI FNIG-NP-NN EVTIAELADA MRRTYARITG
XpUAS       AVEAIARILD RPLE-AQNQI FNIG-NR-NN EVTMRGLAEL MRDVYAEITD
YpUAS       AVDAICRMLD NPEK-ANGHA FNIG-NR-NN EVTMAELADM MRKTYAKITG
RsU4kpxs    GIDALVRIIA NKDGVASGKI YNIG-NP-SN NYSVRELAEM MLKKAGT--I
ArnA        GIEALYRIIE NAGNRCDGEI INIG-NP-EN EASIEELGEM LLASFEKHPL
AtUAS1      AIEAVLLMIE NPER-ANGHI FNVG-NP-NN EVTVRQLAEM MTEVYAKVSG
ZmUAS1      AIEAVMLMIE NPER-ANGQI FNVG-NP-NN EVTVRQLAEM MTEVYSKVSG
PpUAS       AIEAVQKIIE NPAR-ANGHI FNVGNPH-NE VTIQ-ELAEL MTDLYCKISG
NdUAS       AIKAVMLMIE NPER-ANGHI FNVGNPK-NE ATIR-ELAEM MVDVYSRVSG
MvUAS       AIEAVMLMIE NAEE-SNGHI FNVG-NPDNE ASVR-ELANL MTEIYCKVSG
MpUAS       AIDAVLRIIE NPDR-ANMRI FNVGNPM--N EASVKELAEV MTDVYCKISG
hUXS        LVNGLVALMN S--N--VSSP VNLG-NP--E EHTILEFAQL IKNLVG----
MmUXS       LVNGLVALMN S--N--VSSP VNLG-NP--E EHTILEFAQL IKNLVG----
SmUXS       LIDGFVRLMA SPPS--LTGP VNLG-NP--A EFTIGELAEE VIRLTG----
RmUXS       LVDGLILLMN S--D--YSDP VNLG-NP--D EYTIKEFANT IREMVLTPPL
AtUXS3      MVDGLIRLME G--N--DTGP INIG-NP--G EFTMVELAET VKELIN----
Consistency 6966755774 460 6425034544 470 6*8*065034 480 7466288964 490 7445543225 500
```

```
CeUAS       RGQTELPEPI EVSGETFYGE GYD------- DSDRRI---- ---PDITKAQ
GrUAS       DARYNDHPIV VTTADAFYGA GYE------- DCDRRM---- ---PDISKAE
XpUAS       DPRY------ REHPIVEVSS EEF------- YGKGYEDCDR RMP-DLTQAN
YpUAS       DPSYEDYPII EVSSEEFYGE GYE------- DCDRR----- -MP-DISKAK
RsU4kpxs    AEYKE----- NAQKVKLVET TSGAYYGKGY QDVQNR---- --VPKIANTM
ArnA        RHHFP----- PFAGFRVVES SSY------- YGKGYQDVEH RKP-SIRNAH
AtUAS1      EG-AIESPTV DVSSKEFYGE GYD------- DSDKRI---- ---PDMTIIN
ZmUAS1      GK-ILDVPTI DVSSEEFYGE GYD------- DSDKRI---- ---PDMTIIN
PpUAS       TA-RPEVVTV DVPSKEFYGV GYD------- DSDKRI---- ---PEMTQVR
NdUAS       EP-IPETPML DISSQAFYGE GYD------- DSDRRI---- ---PDMVIIQ
MvUAS       KPEPEIPTVD VSSKEFYGEG YDD------- SDKRIP---- ----DMTIIK
MpUAS       KS-KPNLTTI DISSKEFYGE GYE------- DSDRRI---- ---PDMKLVT
hUXS        ---------- SGSEIQFLSE AQD------- DPQKRK---- ---PDIKKAK
MmUXS       ---------- SGSEIQFLSE AQD------- DPQKRK---- ---PDIKKAK
SmUXS       ---------- SRSKIVRRPL PVD------- DPRQRR---- ---PDISLAT
RmUXS       --------SP HNVDIKILPA AED------- DPKKRK---- ---PDITRAK
AtUXS3      ---------- PSIEIKMVEN TPD------- DPRQRK---- ---PDISKAK
Consistency 2101110111 4355445454 4460000000 6456630000 0005875465
```

```
            .........510.........520.........530.........540.........550
CeUAS       QLLGWQPRYN LDQTLEYSMG YWFNGEEGTS WIPQFT---- ----------
GrUAS       RLLGWVPRMS LAEVLYGTMQ SYHDTYT--- APKAA----- ----------
XpUAS       TLLDWYPRRN LRDILLPTMR DYHIR---YA ATSEPEEATQ ----------
YpUAS       ELLGWEPKIA LEDVLLDTMT YFHNE---YA GAMKPAQVAE ----------
RsU4kpxs    EELGWKPTTT MEDTLANIFE AYREH---AA EARSLVD--- ----------
ArnA        RCLDWEPKID MQETIDETLD FFLRT---VD LTDKPS---- ----------
AtUAS1      RQLGWNPKTS LWDLLESTLT YQHRT----- YAEAVKKATS KPVAS-----
ZmUAS1      KQLGWNPKTS LFDLLESTLT YQHKT----- YAEAIKRDIA KSTAS-----
PpUAS       KQLEWEPKTS MYDLMEHTLK YQYSTYA-EA VKKAMSKSTY K---------
NdUAS       RQLGWEPQTP LPDLLEITLK HQYLTY--AA AVKASMA--- ----------
MvUAS       RQLGWEPKTS LPDLLESTLT YQHNT----- YAHAVQQAMC KVLAST----
MpUAS       RQLEWQPTTS LHDLLEFTLA YQHKT----- YSQAVRECVS KTFSTSDL--
hUXS        LMLGWEPVVP LEEGLNKAIH YFRKELEYQA NNQYIPKPKP ARIKKGRTRH
MmUXS       LMLGWEPVVP LEEGLNKAIH YFRKELEYQA NNQYIPKPKP ARVKKGRTRH
SmUXS       EELGWRPKVN LAEGLAHTIR YFDDLLSR-- ---------S MRESAELV--
RmUXS       TYLGWEPKFS VKHGLQETVD WFKSQVAEGA I--------- ----------
AtUXS3      EVLGWEPKVK LREGLPLMEE DFRLRLNVPR N--------- ----------
Consistency 54*7*5*655 8374943764 5544411012 2333222111 1000000000
```

```
            .
CeUAS       -
GrUAS       -
XpUAS       -
YpUAS       -
RsU4kpxs    -
ArnA        -
AtUAS1      -
ZmUAS1      -
PpUAS       -
NdUAS       -
MvUAS       -
MpUAS       -
hUXS        S
MmUXS       S
SmUXS       -
RmUXS       -
AtUXS3      -
Consistency 0
```
