# Supplementary material for: Synthesis of UDP-apiose in Bacteria: The marine phototroph Geminicoccus roseus and the plant pathogen Xanthomonas pisi
Source: PLoS One. 2017 Sep 20;12(9):e0184953. doi: 10.1371/journal.pone.0184953 (PMC5607165; doi:10.1371/journal.pone.0184953)
Supplement: S1 Table — a Chemical shifts are in ppm relative to internal DSS signal set at 0.00 ppm. Proton-proton coupling constants in Hz are shown as well as the J1”, P coupling values between phosphate and the H1” proton of UDP-Api (A), UDP-Xyl (X) and UDP-4-keto-Xyl (K). The chemical shift values for uracil (U) and ribose (R) protons are similar to the uracil and ribose protons of other UDP-sugars. Compounds correspond to structures in Fig 6. * refers to the proton of the branched carbon in UDP-Api and apiofuranosyl-1,2-cyclic phosphate (Ac). Peak assignments were made according to known values [10, 29]. (DOCX) [file pone.0184953.s002.docx]

**S1 Table.**

|  | H1 | H2 | H3*/H3 | H4 | H5 |
| --- | --- | --- | --- | --- | --- |
| **UDP-α-D-Api (A)** |  |  |  |  |  |
| Chemical shifts, δ (ppm^a^),  *peak shape*  J coupling constants (Hz) | 5.71  *Quartet*  J_1”, 2”_ 4.5  J_1”, P_ 5.6 | 4.01  *Quartet*  J_1”, 2”_ 4.5  J_2”, P_ 2.2 | *3.6, 3.6  J_3a”,3b”_ 3.5 | 4.08, 4.09  J_4a”,4b”_ 4.1 |  |
| **UDP-α-D-Xyl (X)** |  |  |  |  |  |
| Chemical shifts, δ (ppm^a^),  *peak shape*  J coupling constants (Hz) | 5.54  *Quartet*  J_1”, 2”_ 3.4,  J_1”, P_ 7.0 | 3.51  *Doublet*  J_2”, 3”_ 3.5 | 3.74  *Doublet*  J_3”, 4”_ 9.5 | 3.67 | 3.78 |
| **UDP-4-keto-Xyl (K)** |  |  |  |  |  |
| Chemical shifts, δ (ppm^a^),  *peak shape*  J coupling constants (Hz) | 5.57  *Quartet*  J_1”, 2”_ 3.5  J_1”, P_ 7.0 | 3.94  *Doublet*  J_2”, 3”_ 5.5 | *3.81  *Doublet*  J_3”, 4”_ 9.8 |  | 3.53 |
| **Apiofuranosyl-1,2-cyclic phosphate (Ac)** |  |  |  |  |  |
| Chemical shifts, δ (ppm^a^),  *peak shape*  J coupling constants (Hz) | 5.93  *Quartet*  J_1”, 2”_ 4.3  J_1”, P_ 15.1 | 4.56  *Doublet*  J_2”, P_ 7.0 | 3.56, 3.6  J_3a”, 3b”_ 12.3 | 3.86, 3.94  J_4a”, 4b”_ 9.4 |  |
| **Ribose (R)** |  |  |  |  |  |
| Chemical shifts, δ (ppm^a^),  J coupling constants (Hz) | 5.97  J_1’, 2’_ 3.6 | 4.35 | 4.34 | 4.26 | 4.19, 4.23  J_5a’, 5b’_ 12 |
| **Uracil (U)** |  |  |  |  |  |
| Chemical shifts, δ (ppm^a^) |  |  |  |  | 5.96 |
| J coupling constants (Hz) |  |  |  |  | J_5, 6_ 8.1 |

**Table 2.**

| Primer | Sequence (5’-3’) |
| --- | --- |
| CeAUS_F | GTATTTTCAGGGCGCCATGAAAATCCTGCTGTTAGGTG |
| CeUAS_R | AGCCGGATCGAATTCACTACGTAAACTGTGGAATCCAAC |
| GrUAS_F | GTATTTTCAGGGCGCCATGCGTGTGGTCATCCTGGGTTG |
| GrUAS_R | AGCCGGATCGAATTCATCAGGCGGCCTTCGGCG |
| XpUAS_F | GTATTTTCAGGGCGCCATGCAGCGAAATCCAATTTCTCAG |
| XpUAS_R | AGCCGGATCGAATTCATCACTGCGTAGCTTCTTCTG |
| pET28b_TEV_F | TGAATTCGATCCGGCTGCTAACAAAGCCCG |
| pET28b_TEV_R | CATGGCGCCCTGAAAATACAGGTTTTC |
| GrRpoD_F | GCATCCCGGTCCACATGATC |
| GrRpoD_R | TCAGGTATCCAGGAAGGAGCG |
| XpSig70_F | GTTGTAGCGATTACCGCCCG |
| XpSig70_R | GCTCAGGCGCAATTTGGC |
