# Supplementary material for: Synthesis of UDP-apiose in Bacteria: The marine phototroph Geminicoccus roseus and the plant pathogen Xanthomonas pisi
Source: PLoS One. 2017 Sep 20;12(9):e0184953. doi: 10.1371/journal.pone.0184953 (PMC5607165; doi:10.1371/journal.pone.0184953)
Supplement: S2 Table — Obtained from IDT. (DOCX) [file pone.0184953.s003.docx]

**S2 Table.**

| Primer | Sequence (5’-3’) |
| --- | --- |
| CeAUS_F | GTATTTTCAGGGCGCCATGAAAATCCTGCTGTTAGGTG |
| CeUAS_R | AGCCGGATCGAATTCACTACGTAAACTGTGGAATCCAAC |
| GrUAS_F | GTATTTTCAGGGCGCCATGCGTGTGGTCATCCTGGGTTG |
| GrUAS_R | AGCCGGATCGAATTCATCAGGCGGCCTTCGGCG |
| XpUAS_F | GTATTTTCAGGGCGCCATGCAGCGAAATCCAATTTCTCAG |
| XpUAS_R | AGCCGGATCGAATTCATCACTGCGTAGCTTCTTCTG |
| pET28b_TEV_F | TGAATTCGATCCGGCTGCTAACAAAGCCCG |
| pET28b_TEV_R | CATGGCGCCCTGAAAATACAGGTTTTC |
| GrRpoD_F | GCATCCCGGTCCACATGATC |
| GrRpoD_R | TCAGGTATCCAGGAAGGAGCG |
| XpSig70_F | GTTGTAGCGATTACCGCCCG |
| XpSig70_R | GCTCAGGCGCAATTTGGC |
